# T_reg_ deficiency‐mediated T_H_1 response causes human premature ovarian insufficiency through apoptosis and steroidogenesis dysfunction of granulosa cells

**DOI:** 10.1002/ctm2.448

**Published:** 2021-06-20

**Authors:** Xue Jiao, Xiruo Zhang, Nianyu Li, Dunfang Zhang, Shidou Zhao, Yujie Dang, Peter Zanvit, Wenwen Jin, Zi‐Jiang Chen, Wanjun Chen, Yingying Qin

**Affiliations:** ^1^ Center for Reproductive Medicine Cheeloo College of Medicine Shandong University Jinan Shandong China; ^2^ Mucosal Immunology Section NIDCR National Institutes of Health Bethesda Maryland USA; ^3^ National Research Center for Assisted Reproductive Technology and Reproductive Genetics Shandong University Jinan Shandong China; ^4^ Key laboratory of Reproductive Endocrinology of Ministry of Education Shandong University Jinan Shandong China; ^5^ Shanghai Key Laboratory for Assisted Reproduction and Reproductive Genetics Shanghai China; ^6^ Center for Reproductive Medicine Ren Ji Hospital School of Medicine Shanghai Jiao Tong University Shanghai China

**Keywords:** apoptosis, granulosa cells, POI, steroidogenesis, T_H_1, T_reg_ cells

## Abstract

Immune dysregulation has long been proposed as a component of premature ovarian insufficiency (POI), but the underlying mediators and mechanisms remain largely unknown. Here we showed that patients with POI had augmented T helper 1 (T_H_1) responses and regulatory T (T_reg_) cell deficiency in both the periphery and the ovary compared to the control women. The increased ratio of T_H_1:T_reg_ cells was strongly correlated with the severity of POI. In mouse models of POI, the increased infiltration of T_H_1 cells in the ovary resulted in follicle atresia and ovarian insufficiency, which could be prevented and reversed by T_reg_ cells. Importantly, interferon (IFN) ‐γ and tumor necrosis factor (TNF) ‐α cooperatively promoted the apoptosis of granulosa cells and suppressed their steroidogenesis by modulating CTGF and CYP19A1. We have thus revealed a previously unrecognized T_reg_ cell deficiency‐mediated T_H_1 response in the pathogenesis of POI, which should have implications for therapeutic interventions in patients with POI.

## Graphical abstract

The Treg cells deficiency with decreased number and impaired suppression function mediate augmented T_H_1 responses in premature ovarian insufficiency (POI). The increased T_H_1 proinflammatory cytokines IFN‐γ and TNF‐α impair steroidogenesis by targeting CYP19A1 and promote apoptosis of granulosa cells partially by down‐regulation of CTGF via JAK‐STAT1 and NF‐κB activation, hence contribute to follicle atresia, ovarian dysfunction and premature insufficiency.

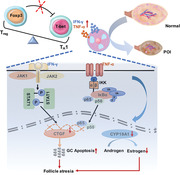

**List of Abbreviations**
bPOI: biochemical POI
CTGF: connective tissue growth factor
CYP19A1: cytochrome P450 family 19 subfamily A member 1
E_2_: estradiol
FOXO1: forkhead transcription factor
FSH: follicle‐stimulating hormone
GATA6: GATA binding protein 6
GCs: granulosa cells
IFN‐γ: interferon‐γ
IL‐10: interleukin‐10
INHBA: inhibin beta A
JAK/ STAT1: janus kinase /signal transducer, activator of transcription 1
MFI: mean fluorescence intensity
NF‐κB: nuclear factor kappa‐B
PBMCs: peripheral blood mononuclear cells
POI: premature ovarian insufficiency
RA: rheumatoid arthritis
SLE: systemic lupus erythematosus
T: testosterone
TGF‐β1: transforming growth factor‐β1
T_H_1: T helper 1
TNF‐α: tumor necrosis factor‐α
T_reg_: regulatory T cells
WT1: Wilms' tumor gene1

## 1 INTRODUCTION

Infertility has increasingly become a public health burden worldwide (∼10%–15%). Premature ovarian insufficiency (POI) is one of the most common causes of female infertility given its inherent feature of compromised reproduction. The disorder is characterized by cessation of ovarian function before the age of 40 years and increased risks of osteoporosis and cardiovascular disease.^1^, ^2^ POI occurs as a continuum of ovarian function decline with progressive menstrual irregularity or amenorrhea, elevated follicle‐stimulating hormone (FSH), and reduced estradiol (E_2_).^3^ It is estimated to affect 1%–5% of reproductive‐aged women. It can result from a small pool of primordial follicles, follicle dysfunction, and premature follicle depletion due to accelerated atresia. POI is highly heterogeneous in etiology, and the majority remains to be elucidated.^4^, ^5^

An autoimmune origin has long been considered to explain 5%–30% of POI cases.^6^, ^7^, ^8^ POI is often associated with concomitant autoimmune diseases, including autoimmune thyroiditis, psoriasis, type 1 diabetes, systemic lupus erythematosus (SLE), and rheumatoid arthritis (RA).^6^, ^9^ POI can also be one component of autoimmune polyendocrinopathy syndrome due to *AIRE* mutations.^10^, ^11^ The presence of oophoritis and circulating autoantibodies has been reported in a subset of women with POI, especially patients with autoimmune adrenal insufficiency.^8^, ^12^, ^13^ Reliable and specific diagnostic markers for autoimmune POI, however, are lacking in the clinic. The mechanisms of autoimmune disturbance underlying ovarian senescence are largely unknown.

Adaptive immune responses are tailored to different types of pathogens through differentiation of naïve CD4^+^ T cells into functionally distinct subsets of effector T cells (T helper 1 [T_H_1], T_H_2, and T_H_17). CD4^+^Foxp3^+^ regulatory T (T_reg_) cells comprise a distinct suppressive lineage and play crucial roles in peripheral immune tolerance.^14^ T_reg_ cell suppressive function can be achieved by direct cell contact through coinhibitory molecules such as CTLA‐4 and the production of immune regulatory cytokines such as transforming growth factor‐β1 (TGF‐β1) and interleukin‐10 (IL‐10).^15^, ^16^ The balance between pro‐ and anti‐inflammatory subsets is finely tuned to maintain immune homeostasis. Quantitative and functional dysregulation of T_reg_ cells or augmented autoreactive response of inflammatory effector T cells underlies the autoimmunity and tissue damage in multiple autoimmune diseases, such as multiple sclerosis, SLE, and RA.^14^ Whether the altered pathogenic T subsets and cytokines, if any, are implicated in the disruption of ovarian microenvironment homeostasis and contribute to the pathogenesis of human POI remain poorly defined.

In this study, we have comprehensively characterized the autoimmune disturbances in patients with POI and demonstrated the augmented T_H_1 autoimmunity and T_reg_ cell deficiency both in the periphery and ovarian microenvironment in POI patients. The decreased ratio of T_reg_ to T_H_1 cells strongly correlated with the severity of POI disease. In experimental POI models in mice, we elucidated the causative role of T_H_1 cells in ovarian damage, which was prevented and suppressed by T_reg_ cells. Importantly, we determined that T_H_1 cytokines interferon (IFN) ‐γ and tumor necrosis factor (TNF) ‐α directly promoted apoptosis and inhibited the proliferation and steroidogenesis of human granulosa cells (GCs) *in vitro* by downregulating the connective tissue growth factor (CTGF) and cytochrome P450 family 19 subfamily A member 1 (CYP19A1). Our results uncovered the augmented T_H_1 response attributed to T_reg_ deficiency in association with ovarian dysfunction in POI, which could provide new insights into autoimmune pathogenesis and clues for novel therapeutic interventions for patients with POI.

## 2 RESULTS

### 2.1 Increased IFN‐γ and TNF‐α in the blood and ovaries of patients with POI

**FIGURE 1:**
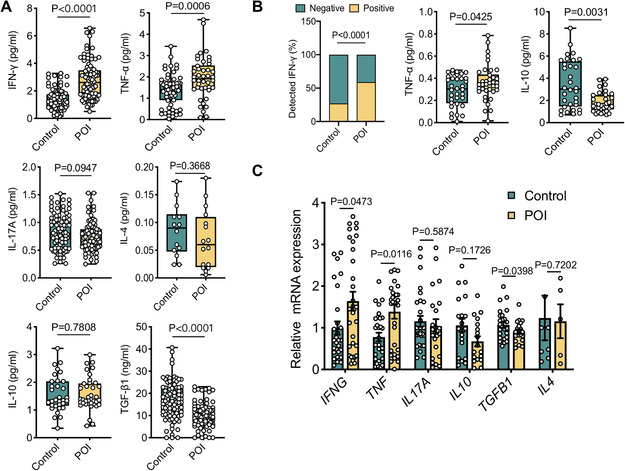
Dysregulated cytokine profile in periphery and ovarian microenvironment in patients with POI. (A) Serum cytokine levels detected by ELISA in control women (*n* = 100) and patients with POI (*n* = 100). Serum IL‐2 could not be detected. (B) Cytokine levels in follicular fluid (FF) detected by ELISA in control women (*n* = 38) and patients with biochemical POI (*n* = 39). IL‐17A, IL‐4, and IL‐2 from FF could not be detected. (C) Quantitative RT‐PCR analysis of cytokines in ovarian granulosa cells in control women (*n* = 31) and patients with biochemical POI (*n* = 31). Data were either shown as scatter plots (mean ± SEM) and analyzed by the unpaired two‐tailed Student's *t*‐test or as box‐and‐whisker plots with analysis of two‐tailed Mann–Whitney U test. Dots represent individual data points. The chi‐square test was used for the positive rates of IFN‐γ from FF

To investigate whether dysregulated immunity occurs in POI, we first determined the serum cytokine profiles in patients with POI (*N* = 100) and control women (*N* = 100) with the respective enzyme linked immunosorbent assays (ELISAs). Interestingly, POI patients showed significantly increased levels of the type 1 proinflammatory cytokines IFN‐γ (*p* < 0.0001) and TNF‐α (*p* = 0.0006) but reduced amounts of the regulatory cytokine TGF‐β1 (*p* < 0.0001) (Figure 1A). No differences were detected for other cytokines, such as IL‐4 (T_H_2), IL‐17A (T_H_17), and IL‐10 (Figure 1A). IL‐2 was undetectable in both controls and patients. To determine whether the dysregulated cytokine profile results from T lymphocytes, we analyzed intracellular cytokines in T cells from peripheral blood mononuclear cells (PBMCs) using flow cytometry. Compared to control women, patients with POI had an increased frequency of CD3^+^IFN‐γ^+^ T cells (*p* = 0.0462), CD3^+^TNF‐α^+^ T cells (*p* = 0.0196), and CD3^+^TNF‐α^+^IFN‐γ^+^ T cells (*p* = 0.0164) (Figure S1). No differences were observed for IL‐17A^+^ and IL‐10^+^ CD3^+^ T cells between the two groups (*p* > 0.05). The percentages of CD4^+^ and CD8^+^ T cells were comparable between POI patients and control subjects (Figure S1). Thus, patients with POI exhibited a systemically augmented T_H_1‐like response.

Given the systemic increase in T_H_1‐type response, we next determined the inflammatory cytokine profile in the ovarian microenvironment by measuring cytokines in follicular fluid (FF) and GCs in patients with biochemical POI (bPOI), which is defined as the early stage of POI and is characterized by decreased follicle quantity or quality^3^ (Figures 1B and 1C; bPOI, *N* = 31; control, *N* = 31). It is impractical to obtain FF or GCs from POI patients because of follicle depletion and ovarian atrophy. Strikingly, we found that women with bPOI already had significantly higher levels of TNF‐α (*p* = 0.0425) in FF than did controls. As some control women and patients showed undetectable levels of IFN‐γ in the FF, we calculated the positive rates of IFN‐γ detection between the two groups and found that there was also a significantly higher frequency of detectable IFN‐γ in bPOI patients than in controls (*p* < 0.0001). Interestingly, patients with bPOI showed reduced amounts of IL‐10 compared to control women (*p* = 0.0031) (Figure 1B). IL‐17A, IL‐4, and IL‐2 levels were undetectable in both patients and controls. In addition, ovarian GCs isolated from women with bPOI showed significantly increased expression of the inflammatory cytokines *IFNG* and *TNF* and decreased *TGFB1* expression compared with the control groups (*p* < 0.05). However, no significant differences were found in *IL17A*, *IL4*, and *IL10* mRNA expression (Figure 1C). The data collectively indicate that patients with early bPOI and overt POI exhibited an increased T_H_1 proinflammatory response in both the periphery and ovarian microenvironments.

**HIGHLIGHTS**

- Deficient T_reg_ cells fail to restrain augmented T_H_1 response in POI patients.
- The increased ratio of T_H_1: T_reg_ cells correlates with severity of POI.
- T_reg_ cells prevent and reverse T_H_1‐mediated ovarian insufficiency in mice.
- T_H_1 cytokines impair GCs growth and steroidogenesis by modulating CTGF and CYP19A1.

### 2.2 T_reg_ cell deficiency in patients with POI

**FIGURE 2:**
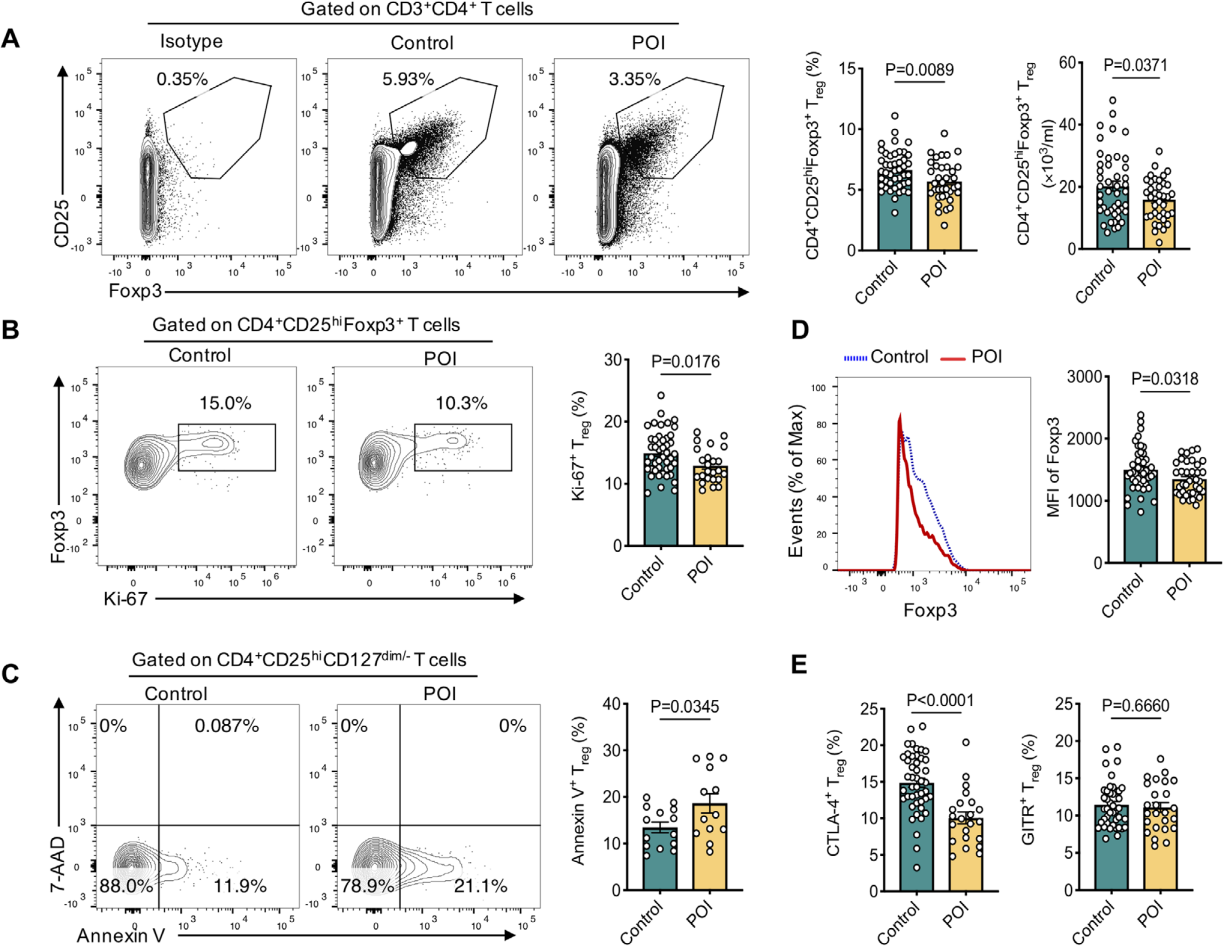
Decreased and functionally impaired CD4^+^CD25^hi^Foxp3^+^ T_reg_ subsets in patients with POI. (A) Representative flow cytometry plots and the statistical analysis of frequency and absolute number of CD4^+^CD25^hi^Foxp3^+^ T_reg_ cells gated on CD3^+^CD4^+^T cells from PBMC in control women (*n* = 45) and patients with POI (n = 37). (B) Representative flow cytometry plots and the statistical analysis of frequency of Ki‐67^+^ cells gated on CD4^+^CD25^hi^Foxp3^+^ T_reg_ cells in control women (*n* = 45) and patients with POI (*n* = 24). (C) Representative flow cytometry plots and the statistical analysis of frequency of Annexin V^+^/7‐AAD^−^cells gated on CD4^+^CD25^hi^CD127^dim/‐^ T_reg_ cells in control women (*n* = 14) and patients with POI (*n* = 13). (D) Representative flow cytometry plots and the statistical analysis of MFI of Foxp3 from CD4^+^CD25^hi^Foxp3^+^ T_reg_ cells in control women (*n* = 45) and patients with POI (*n* = 37). (E) The statistical analysis of frequency of CTLA‐4^+^ cells and GITR^+^ cells gated on CD4^+^CD25^hi^Foxp3^+^ T_reg_ cells in control women (*n* = 45) and patients with POI (*n* = 25). Data were shown as scatter plots (mean ± SEM) and analyzed by unpaired two‐tailed Student's *t*‐test

The abnormal upregulation of T_H_1 cytokines encouraged us to explore whether T_reg_ cell deficiency exists in patients with POI, as T_reg_ cells are a key regulator to control the immune response.^14^, ^17^, ^18^ We first examined the number and phenotype of CD4^+^CD25^hi^Foxp3^+^ T_reg_ cells in PBMCs of patients with POI.^19^ We found that the frequency and absolute number of T_reg_ cells in blood were significantly decreased in women with POI compared with control subjects (Figure 2A, POI, N = 37; control, *N* = 45, *p* = 0.0089; *p* = 0.0371). To understand the mechanisms underlying the decrease in T_reg_ cells, we measured the proliferative rate of T_reg_ cells *ex vivo* with Ki‐67 staining and observed that the fraction of Ki‐67^+^ T_reg_ cells was decreased in patients with POI (Figure 2B, POI, *N* = 24; control, *N* = 45, *p* = 0.0176). In addition, patients with POI had a significantly higher proportion of apoptosis in T_reg_ cells than control women (Figure 2C, POI, *N* = 13; control, *N* = 14, *p* = 0.0345). The data indicate that the decrease in T_reg_ cells in patients with POI is at least partially attributed to their reduced proliferation and increased apoptosis.

We then investigated the suppressive function of T_reg_ cells in POI patients. Given the very limited amounts of blood samples obtained from patients, it was technically impossible to study T_reg_ cell suppression with standard *in vitro* suppressor T cell assays. Instead, we analyzed the expression of Foxp3, CTLA‐4, and GITR, which are indicators of T_reg_ cell function.^16^, ^20^ We found that T_reg_ cells in women with POI exhibited significantly lower levels of Foxp3 expression, as determined by mean fluorescence intensity (Figure 2D, POI, *N* = 37; control, *N* = 45, *p* = 0.0318), and reduced CTLA‐4 positive cells (Figure 2E, POI, N = 22; control, *N* = 45, *p* < 0.0001) compared to control women. However, the GITR expression was comparable between the two groups (Figure 2E, POI, *N* = 25; control, *N* = 42, *p* = 0.6660). Thus, patients with POI show a decreased number and impaired suppressive function of T_reg_ cells, suggesting that a defect in T_reg_ cells might account for the increased levels of proinflammatory cytokines IFN‐γ and TNF‐α in patients with POI.

### 2.3 An increased ratio of T_H_1 cytokines to T_reg_ cells correlates with the severity of ovarian insufficiency in patients

To confirm that the dysregulated ratio of T_H_1:T_reg_ cells is responsible for the severity of ovarian insufficiency, we conducted correlation analyses between inflammatory indicators and ovarian reserve markers in patients with POI (Table 1, Figure S2 and Table S1). As ovarian insufficiency progresses, the E_2_ and testosterone (T) secreted by the ovary gradually decrease, and thus, the pituitary gonadotropin FSH consecutively increases through negative feedback. We found that the amounts of the proinflammatory cytokines IFN‐γ and TNF‐α in the sera had strong positive correlations with FSH (IFN‐γ: FSH, R = 0.36, *p* < 0.001; TNF‐α: FSH, R = 0.43, *p* = 0.002), but negative correlations with E_2_ (IFN‐γ: E_2_, R = ‐0.29, *p* < 0.001; TNF‐α: E_2_, R = ‐0.47, *p* = 0.001). Intriguingly, the level of serum TGF‐β1 negatively correlated with FSH and positively correlated with E_2_ (TGF‐β1: FSH, R = ‐0.37, *p* < 0.001; TGF‐β: E_2_, R = 0.29, *p* < 0.001). Consistently, *TGFB1* mRNA expression in GCs was positively associated with E_2_ (R = 0.33, *p* = 0.04). Significantly, T_reg_ cells exhibited a strong negative correlation with FSH and were positive for E_2_ and T (T_reg_: FSH, R = ‐0.25, *p* = 0.047; T_reg_: E_2_, R = 0.27, *p* = 0.04; T_reg_: T, R = 0.27, *p* = 0.04), suggesting their role in maintaining ovarian reserve and function. Similar correlations were also seen in the ratios of T_reg_:CD3^+^TNF‐α^+^ cells or T_reg_:CD3^+^TNF‐α^+^ IFN‐γ^+^ cells and the levels of FSH, E_2_ and T (*p* < 0.05) (Table 1). Moreover, the negative correlation of FSH with Foxp3 intensity and CTLA‐4 expression further reinforced these associations (Foxp3: FSH, R = ‐0.26, *p* = 0.04; CTLA‐4: FSH, R = ‐0.38, *p* = 0.01). Overall, the correlation analyses suggest a potential causative role of T_H_1/T_reg_ imbalance in the pathogenesis of POI.

**TABLE 1:** Correlation between immune indicators in peripheral with biomarkers of ovarian reserve

Variables	FSH		E2		T	
	R	P	R	P	R	P
serum IFN‐γ	0.36	<0.001	−0.29	<0.001	−0.11	0.15
serum TGF‐β1	−0.37	<0.001	0.29	<0.001	0.12	0.11
serum IL‐17A	−0.003	0.97	0.06	0.39	−0.01	0.91
serum IFN‐γ/TGF‐β1	0.49	<0.001	−0.37	<0.001	−0.11	0.14
serum IL17‐A/TGF‐β1	0.33	<0.001	−0.20	0.01	−0.03	0.66
serum TNF‐α	0.43	0.002	−0.47	0.001	0.01	0.96
serum IL‐10	−0.08	0.52	−0.02	0.87	−0.04	0.77
%T_reg_	−0.25	0.047	0.27	0.04	0.27	0.04
%T_reg_/%CD3^+^TNF‐α^+^	−0.29	0.02	0.29	0.03	0.31	0.02
%T_reg_/CD3^+^IFN‐γ	−0.17	0.20	0.20	0.13	0.23	0.08
%T_reg_/CD3^+^TNF‐α^+^IFN‐γ^+^	−0.33	0.01	0.31	0.02	0.22	0.11
Foxp3 MFI	−0.26	0.04	0.04	0.73	0.06	0.63
%CTLA‐4^+^T_reg_	−0.38	0.01	0.05	0.74	0.08	0.60
%Ki‐67^+^T_reg_	−0.16	0.22	0.09	0.52	0.21	0.13

Data were analyzed by Spearman's correlation.

Abbreviations: E2, Estradiol; FSH, follicle stimulating hormone; T, testosterone.

### 2.4 T_reg_ cells ameliorate experimental POI by suppressing the T_H_1 response

**FIGURE 3:**
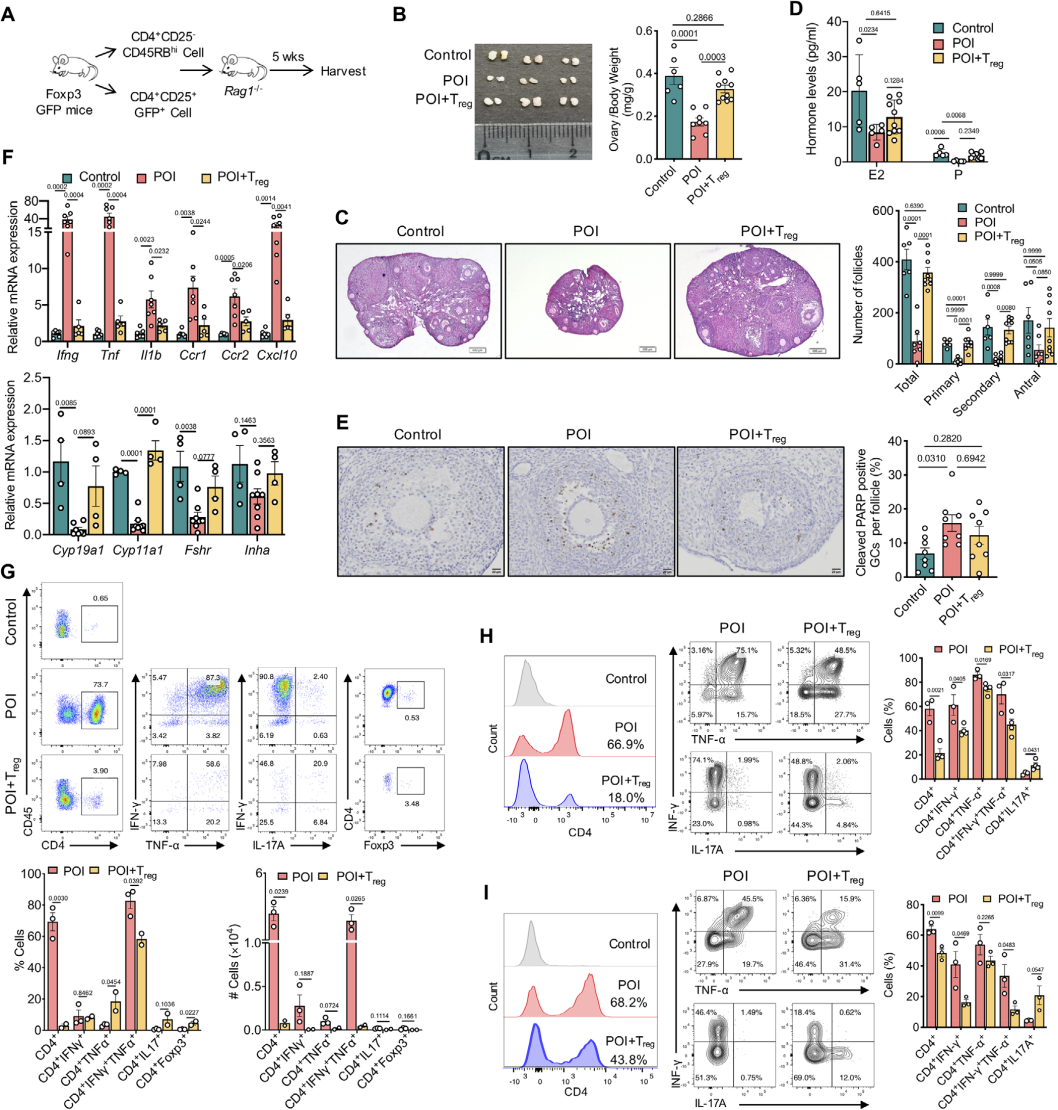
T_reg_ cells ameliorate experimental POI by suppressing T_H_1 response. (A) The experimental scheme of the adoptive transfer model. (B) The representative ovary gross photos and statistics of ovary/body weight ratio in control (*n* = 6), POI (*n* = 8), and POI+T_reg_ (*n* = 10) group. (C) Representative histology images of ovary sections (scale bars, 600 μm) and quantification of ovarian follicles in three groups. (D) Serum estradiol and progesterone level detected by radioimmunology in three groups. (E) Representative immunohistochemical images and statistics of cleaved‐PARP in ovarian granulosa cells (scale bars, 20 μm). (F) Gene expression of inflammatory cytokines and chemokines, and genes related to ovarian function by qRT‐PCR in ovaries. (G) Representative flow cytometry plots of infiltrated CD4^+^ T cells in ovary in three groups and statistics of CD4^+^ T cells and different subsets in POI and POI+T_reg_ groups. The ovarian cells from two mice were pooled together as one sample for FACS. (H) Representative flow cytometry plots and statistics of CD4^+^ T cells and different subsets in spleens in POI and POI+T_reg_ group. (I) Representative flow cytometry plots and statistics of CD4^+^ T cells and different subsets in ovary‐draining lymph nodes in POI and POI+T_reg_ group. The experiments were repeated for three times, and data from one representative experiment were shown. All data were shown as scatter plots (mean ± SEM) and analyzed by one‐way ANOVA test (B‐F) and unpaired two‐tailed Student's *t*‐test (G‐I)

We next determined the role of T_H_1 cell‐mediated inflammation in the pathogenesis of ovarian insufficiency and the regulatory function of T_reg_ cells in suppressing T_H_1 cells in experimental POI models in mice. First, we utilized a classic model of colitis induced by adoptive transfer of normal CD4^+^CD25^−^45RB^hi^ T cells into *Rag 1^−/−^* recipient mice,^21^ which also induced ovarian insufficiency mimicking human POI. The function of T_reg_ cells was determined by cotransfer of CD4^+^CD25^+^GFP^+^ cells isolated directly from Foxp3‐GFP transgenic mice (experimental scheme in Figure 3A). After 5 weeks, *Rag1* ^−/‐^ mice transferred with CD4^+^CD25^−^CD45RB^hi^ T cells exhibited the ovarian insufficiency phenotype, with smaller ovarian size and decreased number of follicles in different stages (POI group, Figures 3B and 3C). The levels of estradiol and progesterone were also markedly decreased (Figure 3D). As excessive apoptosis of GCs is recognized as one of the important mechanisms in premature follicle atresia and depletion,^22^, ^23^ we analyzed GC apoptosis in ovaries with immunohistochemical staining of cleaved PARP. We found that the proportion of cleaved PARP‐positive cells per follicle was much higher in the POI group, and the apoptotic signals were specifically distributed in the GCs of growing antral follicles, indicating increased apoptosis of GCs in growing follicles associated with ovarian dysfunction and POI (Figure 3E). Importantly, increased gene expression of proinflammatory cytokines (*Ifng*, *Tnf*, and *Il1b*) and chemokines (*Ccr1*, *Ccr2*, and *Cxcl10*), and decreased expression of genes related to ovarian function (*Cyp19a1*, *Cyp11a1*, and *Fshr*) were observed in the ovaries of mice receiving CD4^+^CD25^−^45RB^hi^ T cells (Figure 3F). Flow cytometry analysis of single cells in ovaries revealed massive infiltration of lymphocytes predominated by CD4^+^IFN‐γ^+^TNF‐α^+^ T cells, whereas IL‐17A^+^ T_H_17 cells and Foxp3^+^ T_reg_ cells were virtually absent, suggesting a key role of T_H_1 cells in the disease (Figure 3G). In contrast, mice receiving cotransferred T_reg_ cells (POI+T_reg_) exhibited little or no infiltration of lymphocytes in ovaries, which was similar to the unmanipulated control mice (Figure 3G). Cotransfer of T_reg_ cells effectively prevented ovarian weight loss, improved ovarian function, reduced the amounts of proinflammatory cytokines in the ovary and decreased GC apoptosis (Figures 3B‐3E). Consistently, the mRNA expression of the genes that reflected ovarian function, including *Cyp19a1, Cyp11a1*, and *Fshr*, was also augmented, which was accompanied by a reduction in the mRNA expression of cytokines and chemokines (Figure 3F). The data demonstrated a dramatic amelioration of ovarian insufficiency following T_reg_ cell cotransfer. The number of IFN‐γ‐ and TNF‐α‐producing CD4^+^ T cells was also reduced in the ovary (Figure 3G), spleen and draining lymph nodes (Figures 3H and 3I). The data collectively indicate a key role of T_reg_ cells in suppressing the pathogenic function of T_H_1 inflammation in the ovary.

### 2.5 T_reg_ cell depletion exacerbates ovarian insufficiency by increasing T_H_1 cells in the ovary

**FIGURE 4:**
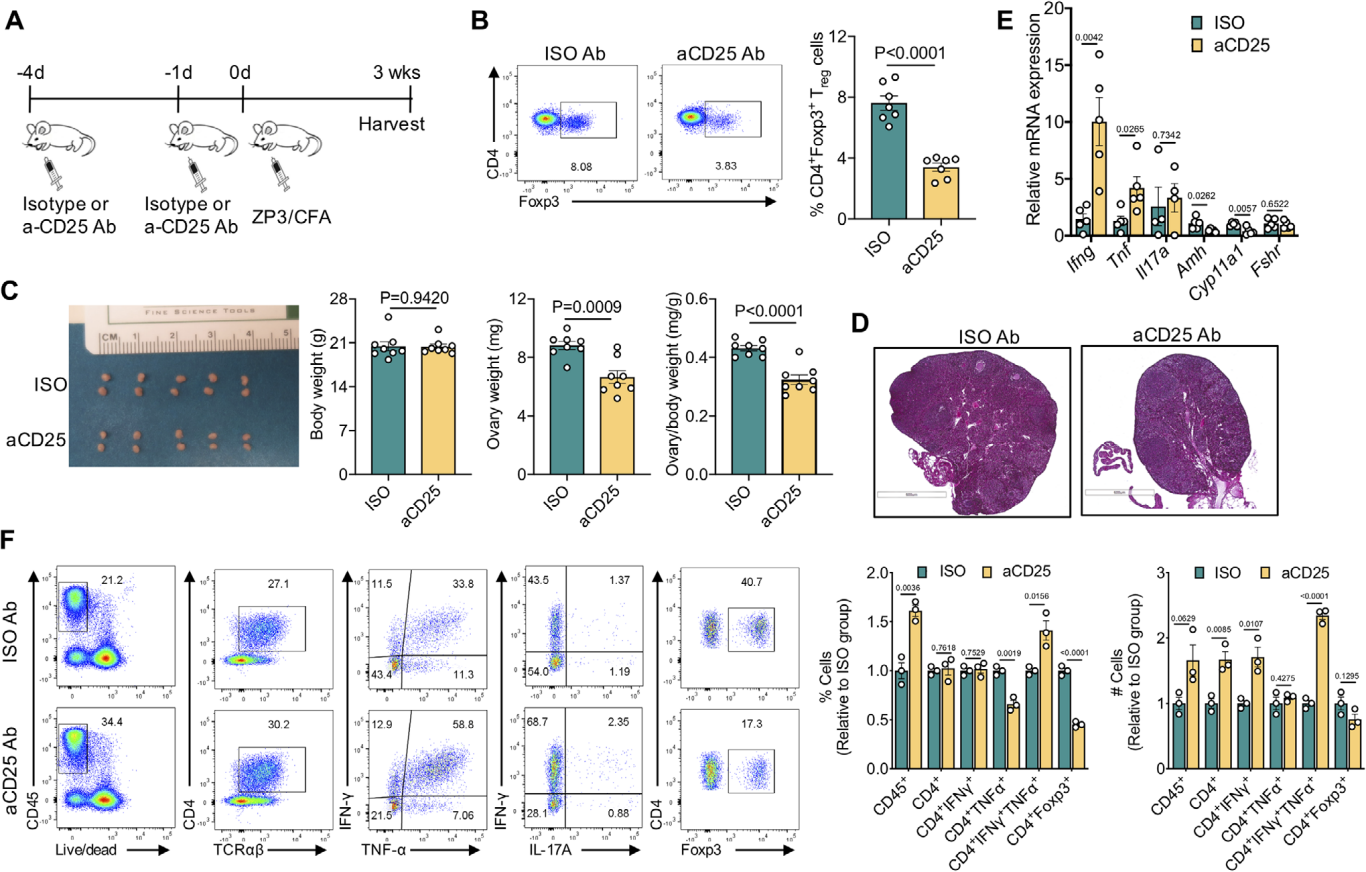
T_reg_ depletion aggravated premature ovarian insufficiency in mice. (A) The experimental scheme of T_reg_ depletion in experimental POI model. (B) Representative flow cytometry plots and statistics of percentage of T_reg_ cells in PBMC of the isotype antibody (ISO Ab) and anti‐CD25 antibody (aCD25 Ab) treated groups on day 0. (C) Representative photo of gross ovary and statistics of body weight, ovary weight and ovary/body weight ratio in ISO Ab and aCD25 Ab group. (D) Representative histology images of ovary sections (scale bars, 600 μm) in ISO Ab and aCD25 Ab group. (E) Gene expression of inflammatory cytokines and factors related to ovarian function by qRT‐PCR in the ovaries of ISO Ab and aCD25 Ab group. (F) Representative flow cytometry plots and statistics of percentage (fold change relative to ISO Ab group) and absolute number (fold change relative to ISO Ab group) of infiltrated immune cells in ovary in ISO Ab and aCD25 Ab group. The ovarian cells from two mice were pooled together as one sample for FACS. The experiments were repeated for three times, and data from one representative experiment were shown. All data were expressed as the mean ± SEM and analyzed by the unpaired two‐tailed Student's *t*‐test

To further validate the regulatory function of T_reg_ cells in experimental POI, we depleted endogenous T_reg_ cells with anti‐CD25 antibody (PC61) in another model of POI induced by immunization with Zp3 peptide emulsified in CFA^24^ (experimental scheme in Figure 4A). Before ZP3/CFA immunization, more than 50% of CD4^+^Foxp3^+^ T_reg_ cells were effectively depleted in peripheral blood by anti‐CD25 antibody administration (Figure 4B). After 3 weeks, we found that the mice injected with anti‐CD25 antibody had ovaries that were more atrophic, with smaller ovarian size, decreased ovarian weight, premature follicle depletion and ovarian fibrosis (Figures 4C and 4D). Decreased expression of genes related to ovarian steroidogenesis and function (*Amh* and *Cyp11a1*) was also detected in ovaries of the anti‐CD25 antibody group (Figure 4E). Flow cytometry analysis in ovaries revealed substantially increased infiltration of IFN‐γ‐ and TNF‐α‐producing T_H_1 cells and a marked decrease of CD4^+^Foxp3^+^ T_reg_ cells in the anti‐CD25 antibody group (Figure 4F). The increased T_H_1 inflammation after T_reg_ cell depletion in ovaries was further confirmed by the significantly increased mRNA expression of the proinflammatory cytokines *Ifng* and *Tnf* (Figure 4E). The data collectively indicate that T_reg_ cells play a key role in preventing and suppressing experimental POI by inhibiting T_H_1 cells.

### 2.6 IFN‐γ and TNF‐α impair cell growth and steroidogenesis of human granulosa cells in culture

**FIGURE 5:**
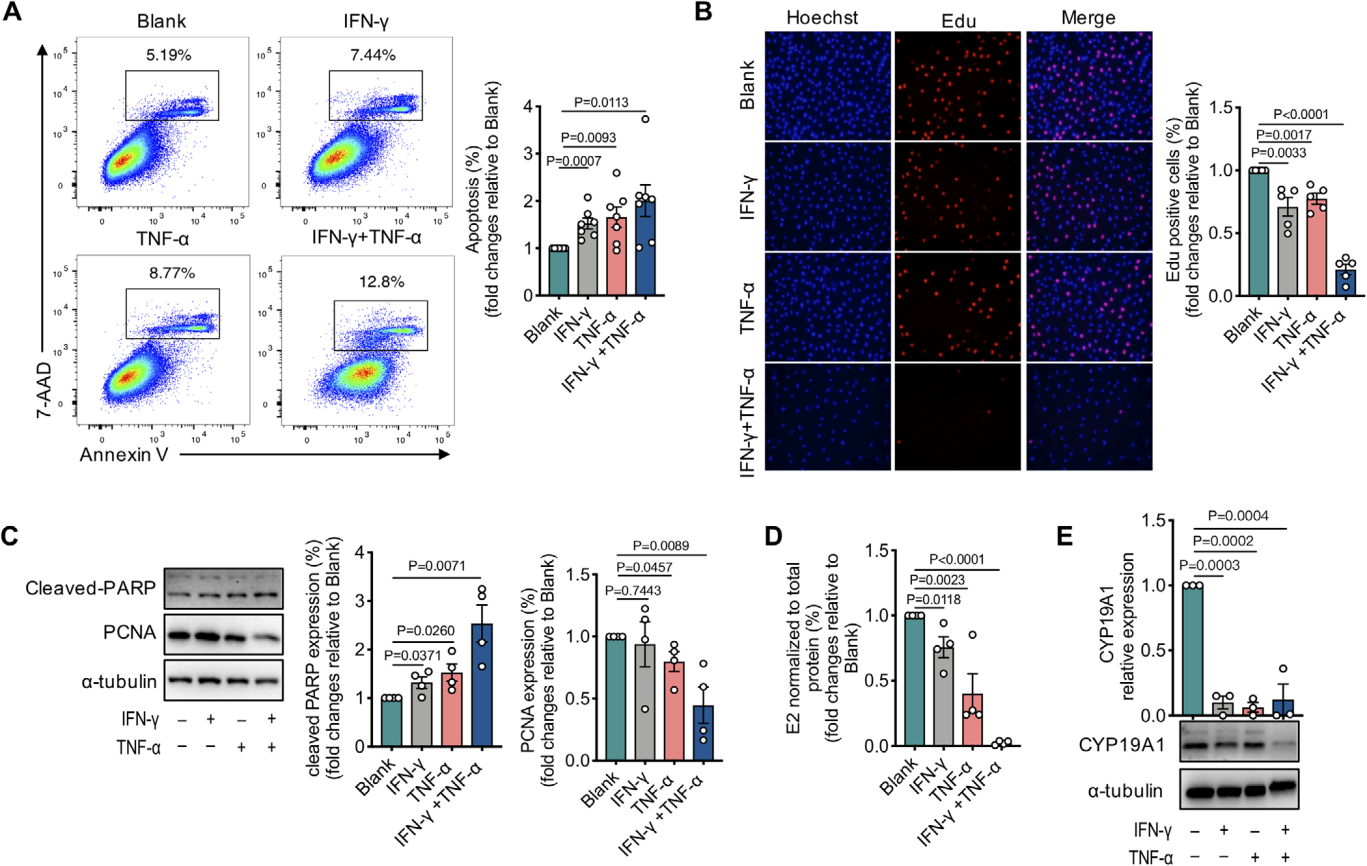
IFN‐γ and TNF‐α exposure impair granulosa cell growth and steroidogenesis *in vitro*. (A) Representative flow cytometry plots and the statistics of frequency of Annexin V/7‐AAD double positive cells. (B) Representative immunofluorescent microscopy images (scale bars, 150 μm) and the statistics of Edu positive cells. (C) Cleaved‐PARP and PCNA protein levels detected by western blot, and protein quantification was analyzed by being normalized to α‐tubulin. (D) Estradiol production measured by chemiluminescence. (E) The expression of CYP19A1 mRNA and protein levels by qRT‐PCR and Western blot. Data were presented relative to the control group. The results were expressed as mean ± SEM from at least three independent experiments. Data were analyzed by the one‐way ANOVA test

Having observed the positive correlation between T_H_1 cytokines and the severity of POI and validated the key pathogenic role of these cytokines in experimental POI *in vivo*, we next investigated the functional impact of T_H_1 cytokines on human ovarian GCs *in vitro*. We cultured human KGN cells in the presence of rhIFN‐γ (50.0 ng/ml) and rhTNF‐α (50.0 ng/ml) either alone or in combination for 48 h and measured their apoptosis and proliferation. We found that IFN‐γ and TNF‐α induced profound increase in cell apoptosis and a decrease in the proliferation of human KGN cells (Figures 5A and 5B). Consistently, both cytokines substantially increased cleaved PARP but decreased PCNA expression, indicating that IFN‐γ and TNF‐α could impair cell growth by promoting apoptosis and decreasing proliferation (Figure 5C). It is well known that the synthesis and secretion of estrogen is the principal endocrine function of GCs and is mediated by the critical rate‐limiting enzyme CYP19A1 aromatase preferentially expressed in GCs.^25^ We found that estradiol production by human KGN cells was significantly impaired upon IFN‐γ and TNF‐α treatment (Figure 5D). Meanwhile, IFN‐γ and TNF‐α treatment significantly decreased the mRNA and protein expression of CYP19A1 (Figure 5E). Importantly, synergistic reduction in GC growth, and steroidogenesis was observed with the combination of IFN‐γ and TNF‐αcompared to either cytokine alone (Figures 5A‐5E). Taken together, these data indicate that IFN‐γ and TNF‐α directly result in granulosa cell dysfunction and thus contribute to follicle atresia and ovarian insufficiency.

### 2.7 A role of CTGF in T_H_1 cytokine‐induced granulosa cell apoptosis

**FIGURE 6:**
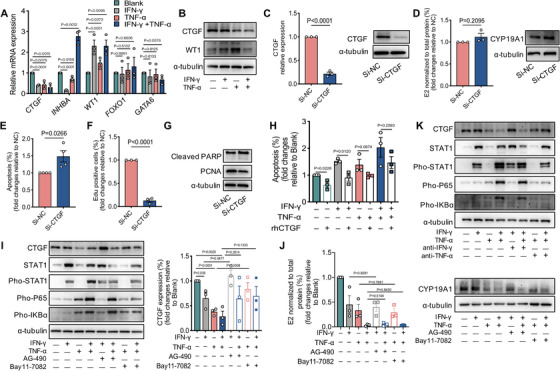
The role of CTGF in IFN‐γ and TNF‐α‐induced granulosa cell apoptosis and steroidogenesis. (A and B) The human KGN cells were treated in the absence or presence of rhIFN‐γ (50.0 ng/ml), rhTNF‐α (50.0 ng/ml) or a combination for 48 hours. (A) Expression of different markers related to granulosa cell function analyzed by qRT‐PCR. (B) Expression of CTGF and WT1 protein detected by western blot. (C‐G) The human KGN cells were transfected with 50 nM CTGF siRNA (Si‐CTGF) and 50 nM control siRNA (Si‐NC) for 48 h to silence endogenous *CTGF* expression. (C) The efficiency of sh‐CTGF was confirmed by qRT‐PCR (left) and western blot (right). (D) Estradiol production was measured by Chemiluminescence (left) and CYP19A1 protein level detected by western blot (right). (E) Statistics of the percentage of Annexin V/7‐AAD double positive cells. (F) Statistics of the percentage of Edu positive cells. (G) Cleaved‐PARP and PCNA protein level detected by western blot. (H) The human KGN cells were cultured with rhCTGF (20.0 ng/ml) in the presence of rhIFN‐γ (50.0 ng/ml), rhTNF‐α (50.0 ng/ml), or a combination for 48 h. Statistics of frequency of Annexin V/7‐AAD double positive cells. (I‐J) The human KGN cells were treated with or without 10 μM inhibitor of JAK/STAT1(AG‐490) and 5 μM inhibitor of NF‐κB (Bay11‐7082) for 1 h prior to cytokines stimulation. (I) The expression of CTGF, STAT1 and p‐STAT1, p‐P65, p‐IKBα was detected by western blot (left). CTGF protein quantification was analyzed by being normalized to α‐tubulin (right). (J) Estradiol production was measured by Chemiluminescence (left) and normalized to the control group; CYP19A1 protein level was examined using western blot (right). (K) The human KGN cells were treated with 1 μg/ml neutralizing antibody for IFN‐γ and TNF‐α for 1 h followed by treatment with cytokines. The expression of CTGF, STAT1 and p‐STAT1, p‐P65, p‐IKBα was detected by western blot. Data were presented relative to the control group. The results were expressed as mean ± SEM from at least three independent experiments. Data were analyzed by the one‐way ANOVA test (A and H‐J) or unpaired two‐tailed Student's *t*‐test (C‐F)

We next investigated the molecular mechanisms downstream of the effects of IFN‐γ and TNF‐α on GCs. A number of functional signature genes of GCs including *CTGF*, inhibin beta A (*INHBA*), Wilms' tumor gene 1 (*WT1*), the forkhead transcription factor (*FOXO1*), and GATA binding protein 6 (*GATA6*), were first examined by RT‐qPCR. We found significantly decreased *CTGF* and *INHBA* but increased *WT1* mRNA expression in cultures after IFN‐γ and TNF‐α exposure (Figure 6A). Given the contradictory effect of IFN‐γ plus TNF‐α treatment compared with IFN‐γ or TNF‐α alone on *INHBA* expression, we then focused on the protein expression of CTGF and WT1 after cytokine exposure. Interestingly, only CTGF exhibited consistent downregulation at both the mRNA and protein levels (Figure 6B). To further determine whether the effects of IFN‐γ and TNF‐α on GCs were mediated by CTGF, we downregulated endogenous *CTGF* expression by employing shRNA transfection in KGN cells (Figure 6C). We found that CTGF reduction had no effect on estradiol synthesis or *CYP19A1* expression in KGN cells in response to IFN‐γ and TNF‐α treatment (Figure 6D). However, downregulation of *CTGF* significantly enhanced the apoptosis and suppressed the proliferation of KGN cells, which was further evidenced by increased cleaved PARP expression and decreased PCNA expression (Figures 6E‐6G). Conversely, the addition of exogenous rhCTGF to KGN cells effectively rescued the cell apoptosis induced by both cytokines (Figure 6H). These data indicate that CTGF deficiency is critical for T_H_1 cytokine‐induced growth impairment in granulosa cells.

### 2.8 IFN‐γ and TNF‐α downregulate CTGF through JAK‐STAT1 and NF‐κB activation

**FIGURE 7:**
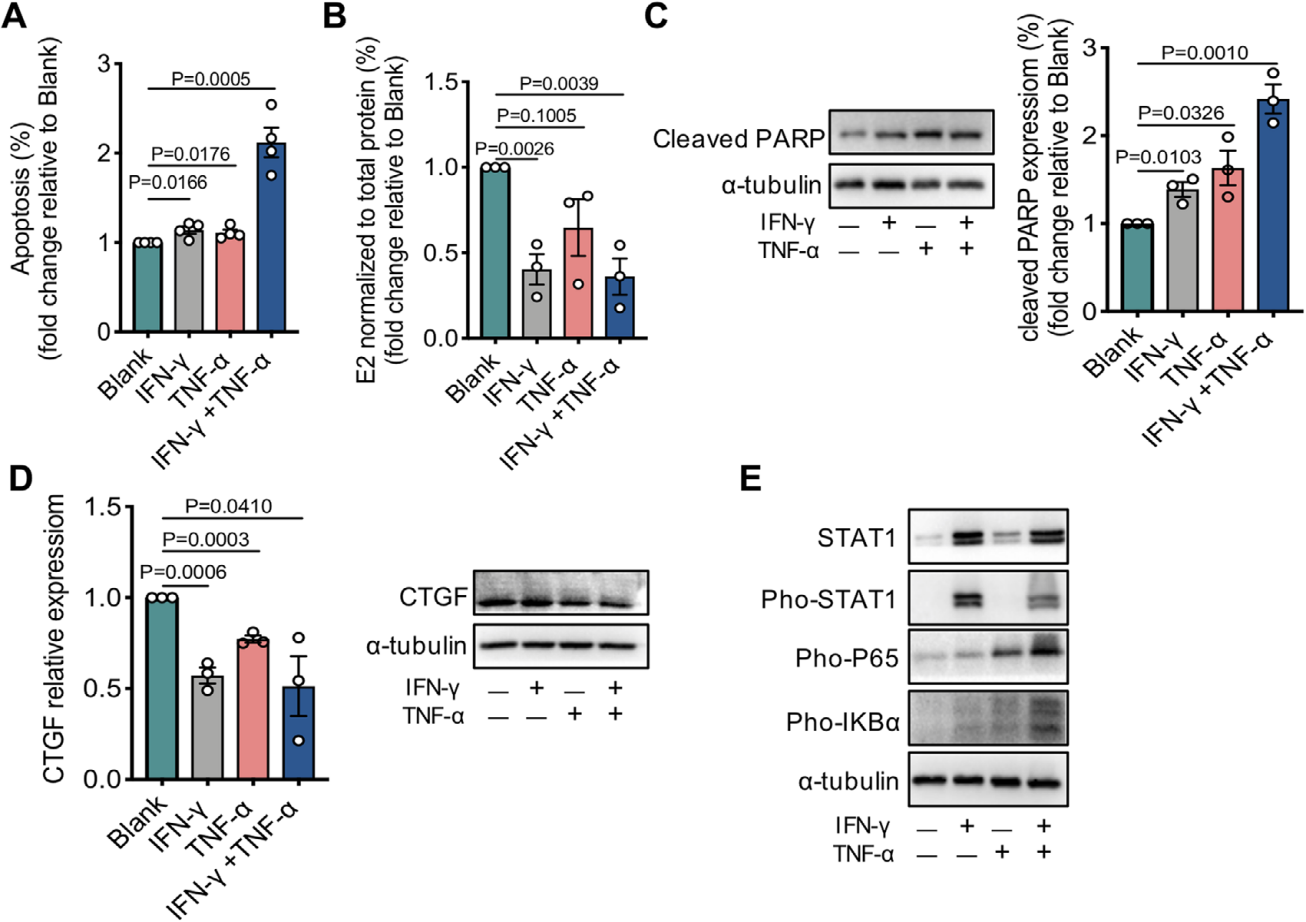
T_H_1 cytokines impair growth and steroidogenesis of mouse primary granulosa cells (mGCs). The mGCs were treated in the absence or presence of rmIFN‐γ (50.0 ng/ml), rmTNF‐α (50.0 ng/ml) or a combination for 48 h. (A) The statistics of frequency of Annexin V/7‐AAD double positive cells. (B) Estradiol production measured by Chemiluminescence. (C) The expression of cleaved‐PARP detected by western blot (left), and cleaved‐PARP protein quantification was analyzed by being normalized to α‐tubulin (right). (D) The expression of CTGF analyzed by qRT‐PCR (left) and western blot (right). (E) The expression of STAT1 and p‐STAT1, p‐P65, p‐IKBα was examined using western blot. Data were presented relative to the control group. The results were expressed as mean ± SEM from at least three independent experiments and each performed in triplicate. Data were analyzed by the one‐way ANOVA test

We next explored how IFN‐γ and TNF‐α regulated CTGF expression. The janus kinase (JAK)/signal transducer and activator of transcription‐1 (STAT1) and nuclear factor kappa‐B (NF‐κB) signaling was activated by IFN‐γ and TNF‐α, as evidenced by increased phosphorylation of STAT1, IKBα, and p65 in human KGN cells (Figure 6I). The addition of inhibitors of JAK or IKBα phosphorylation attenuated IFN‐γ‐ and TNF‐α‐induced inhibitory effects on CTGF expression in KGN cells (Figure 6I). CTGF expression was also reversed when using neutralizing antibodies against IFN‐γ and TNF‐α (Figure 6K). However, the suppression of E_2_ synthesis by IFN‐γ and TNF‐α could not be reversed by either JAK/STAT1 or NF‐κB inhibitors (Figure 6J). Similar results were obtained in murine primary GCs in cultures (Figure 7). These data indicate that IFN‐γ and TNF‐α downregulate CTGF in granulosa cells via JAK‐STAT1 and NF‐κB activation.

## 3 DISCUSSION

**FIGURE 8:**
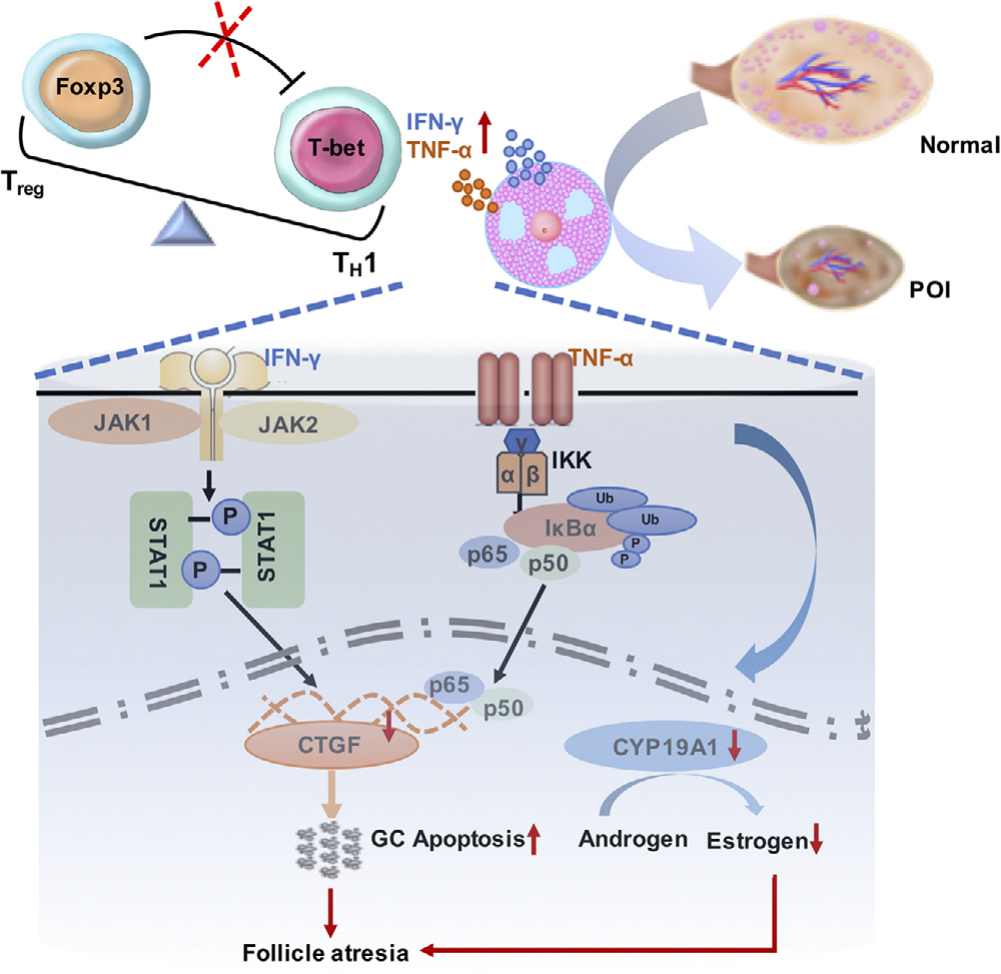
The proposed working model of POI. The T_reg_ cells deficiency with decreased number and impaired suppression function could mediate augmented T_H_1 responses in premature ovarian insufficiency (POI). The increased T_H_1 proinflammatory cytokines IFN‐γ and TNF‐α impair steroidogenesis by targeting CYP19A1 and promote apoptosis of granulosa cells partially by down‐regulation of CTGF via JAK‐STAT1 and NF‐κB activation, hence contribute to follicle atresia, ovarian dysfunction and premature insufficiency

Here for the first time we have comprehensively characterized the phenotype and function of immune responses in human ovarian insufficiency. Our data provide compelling evidence that patients with POI have decreased and functionally impaired CD4^+^CD25^hi^Foxp3^+^ T_reg_ cells and increased T_H_1‐dominant inflammation in both the periphery and ovarian microenvironments. This T_reg_:T_H_1 disturbance and altered inflammatory cytokine profile were strongly correlated with progression of human ovarian insufficiency, and the potentially causative effects were validated in experimental POI in mice. The increased IFN‐γ and TNF‐α impair steroidogenesis by targeting CYP19A1 and promote apoptosis of GCs in part by downregulating CTGF via JAK‐STAT1 and NF‐κB activation, hence contributing to follicle atresia, ovarian dysfunction, and premature insufficiency (proposed model, Figure 8).

The immune system is critical for optimal ovarian homeostasis and reproductive function.^26^, ^27^ However, the pathogenic functions of the immune cells in POI have not been clearly elucidated. Here, we revealed that the T_H_1‐like cytokines, particularly IFN‐γ and TNF‐α, may contribute to the pathogenesis of POI. Evidence supporting this conclusion included selectively systemic and ovarian increases in the proinflammatory cytokines TNF‐α and IFN‐γ and related T_H_1 cells. Intriguingly, other T cell subsets such as T_H_2 and T_H_17 cells and their signature cytokines were not found to change in POI patients. This suggests that POI is likely a T_H_1‐mediated autoimmune disorder.

In exploring the underlying mechanisms for the preferential increase in T_H_1‐like proinflammatory cytokines in POI, we discovered that deficiency in the number and function of T_reg_ cells might play a key role. Several findings supported this conclusion. Although a decrease in CD4^+^CD45RA^−^Foxp3^hi^ effector T_reg_ cells was reported in POI patients,^28^ the detailed phenotype and functional relevance of T_reg_ cells in maintaining ovarian function were still unclear. We have revealed that the decrease in T_reg_ cells was attributable to their reduced proliferation and increased apoptosis in POI patients. Given the lack of suitable and validated markers to distinguish naturally occurring T_reg_ cells and induced T_reg_ cells in complex contexts in humans, no further subtyping was explored here. Importantly, we uncovered that T_reg_ cells in POI patients displayed reduced Foxp3 and CTLA‐4 expression, which accounts for the compromised suppressive ability of T_reg_ cells. In addition, the decreased inhibitory cytokines IL‐10 and TGF‐β may also contribute to the increased T_H_1‐like inflammatory cytokines in POI patients, although the cellular sources of these regulatory cytokines remain unknown. More importantly, the strong correlations between IFN‐γ, TNF‐α, or T_reg_ cells and markers of ovarian function further support that the regulation of T_H_1‐like inflammation by T_reg_ cells contributes to immune homeostasis in the ovary and the maintenance of ovarian function.

Determining the effect of the T_reg_ cell deficiency‐mediated increase in T_H_1 inflammation on ovarian insufficiency is of great importance to clarify the pathogenesis of POI. By using two different animal models of experimental POI, we confirmed the causative role of IFN‐γ and TNF‐α cytokines in POI mice and elucidated the key function of T_reg_ cells in controlling T_H_1‐like inflammatory responses. In *Rag1^−/−^* mice that were adoptively transferred with CD4^+^CD25^−^CD45RB^hi^ T cells, a massive infiltration of immune cells, predominated by IFN‐γ, and TNF‐α‐producing CD4^+^ T cells, was observed in inflamed ovaries. These mice exhibited the phenotype of ovarian insufficiency. Of note, the apoptosis of GCs was preferentially distributed in the growing follicles, consistent with previous reports on oophoritis, in which the immune response was privileged mainly in antral and growing follicles.^29^ These data indicate that augmented T_H_1 response with IFN‐γ and TNF‐α is the major force that induces accelerated follicle atresia. Support for this claim also came from the fact that cotransfer of T_reg_ cells significantly restrained the T_H_1 effector cell response in the ovary and consequently alleviated ovarian damage and greatly restored ovarian function. In contrast, T_reg_ cell depletion in ZP3‐induced POI mice resulted in exacerbated activation and expansion of CD4^+^ T cells and the production of T_H_1 cytokines in the ovary and consequently aggravated ovarian atrophy. These findings provide compelling evidence that T_H_1‐like inflammatory cytokines play a deleterious role in the ovarian microenvironment in POI, which is controlled primarily by the number and intact function of T_reg_ cells.

The follicular microenvironment is critical for folliculogenesis and the acquisition of oocyte competence.^30^ A cascade of intraovarian/perifollicular cytokines and chemokines could mediate communication among lympho‐hemopoietic cells, somatic cells and oocytes by autocrine or paracrine action.^26^, ^27^ Having demonstrated increased IFN‐γ and TNF‐α in POI patients and experimental POI mouse models, we further clarified that IFN‐γ and TNF‐α directly affected the GC growth and steroidogenesis. Exposure to IFN‐γ or TNF‐α profoundly induced apoptosis and suppressed proliferation and thus impaired GC growth. In addition, both cytokines downregulated the key enzyme CYP19A1 aromatase and consequently decreased E_2_ levels. Importantly, estradiol contributes to GC proliferation and follicle differentiation as an intraovarian regulator in folliculogenesis.^31^ Therefore, dysregulation of steroidogenesis in GCs might aggravate the apoptosis induced by IFN‐γ and TNF‐α exposure, and vice versa. Taken together, these results indicated that T_H_1 inflammatory cytokines induce GC apoptosis and dysfunction and contribute to follicle atresia.

CTGF, highly expressed in the granulosa cells of growing follicles as an autocrine/paracrine factor, is a critical regulator of granulosa cell differentiation, follicle growth, tissue remodeling, and ovulation involved in folliculogenesis.^32^, ^33^ Of the crucial genes related to GC function, CTGF was found to be one of the core targets given its significant and consistent changes at both the transcriptional and translational levels after IFN‐γ and TNF‐α exposure. We have revealed that the proapoptotic effect of IFN‐γ and TNF‐α on GCs is mediated by CTGF downregulation, evidenced by increased apoptosis and decreased proliferation after *CTGF* silencing and by reduced apoptosis after rhCTGF treatment. This was consistent with the findings in granulosa cell‐specific *Ctgf* deficiency in mice, which showed increased GC apoptosis, disrupted follicular development and reduced fertility.^34^ It has been reported that IFN‐γ and TNF‐α could reduce CTGF promoter activity and decrease its expression via the STAT1 and NF‐κB pathways in dermal fibroblasts, pancreatic stellate cells, and lung endothelial cells.^35^, ^36^, ^37^ However, the modulation of CTGF by both cytokines in GCs is unclear. We found that after exposure to IFN‐γ and TNF‐α, JAK‐STAT1 and NF‐κB signaling were activated with increased expression of p‐STAT1, p‐IKBα, and p‐P65 in GCs. With JAK inhibitors, IKBα phosphorylation inhibitors or IFN‐γ /TNF‐α neutralization, the effect of both cytokines on CTGF downregulation was attenuated, indicating that the JAK‐STAT1 and NF‐κB pathways participate in the regulation of IFN‐γ and TNF‐α on CTGF in human GCs. The data provide the mechanism by which IFN‐γ and TNF‐α promote granulosa cell apoptosis, at least partially by downregulating CTGF through the JAK‐STAT1 and NF‐κB pathways, respectively.

Of note, estrogen has been extensively studied for its immunomodulatory role in different immune responses.^38^, ^39^ Generally, low E_2_ concentrations promote T_H_1‐type responses and increase IFN‐γ production, whereas high E_2_ levels augment T_H_2‐type responses.^40^, ^41^, ^42^ In addition, exogenous E_2_ could drive T_reg_ expansion and enhance the conversion of naïve CD4^+^CD25^−^ T cells to CD4^+^CD25^+^ T_reg_ cells with increased Foxp3 expression.^43^, ^44^, ^45^ We have revealed that the T_reg_ cell deficiency‐mediated increase in T_H_1 inflammation impaired steroidogenesis in GCs, which might account for the low estrogen in patients with POI. Meanwhile, the low estrogen status would also restrain T_reg_ cell number and function so that T_reg_ cells could not efficiently suppress T_H_1 inflammation. Consistently, an increase in proinflammatory cytokines, such as IL‐1β, IFN‐γ, TNF‐α, and MCP‐1 has also been reported in the post‐menopausal women.^46^ Therefore, the long‐term estrogen deficiency in POI patients might facilitate the skewing of immune tolerance toward T_H_1 immunity and in turn underlie the exacerbation of ovarian insufficiency. The mutual interaction between hormone dysregulation and immune disturbance result in an extreme negative feedback loop, ultimately leading to the progression of ovarian insufficiency. It is postulated that low estrogen status might also confer greater susceptibility and even participate in the onset of concomitant autoimmune diseases with POI.

Currently, there remains no effective strategy to ameliorate ovarian function and fertility for patients with POI. Typically, these women ultimately pursue egg donation or adoption. The POI patients with autoimmune disturbance usually have residual follicles and might benefit from early immune intervention.^47^, ^48^ Our data quantifying the decreased number and functional impairment of T_reg_ cells in patients with POI and the effectiveness of T_reg_ adoptive transfer in murine POI suggest a potential for T_reg_‐mediated treatment in the clinic. Hopefully, with efforts in T_reg_ cell engineering to enhance their specificity, stability, and functional activity, T_reg_ cell therapy will become a practical method for POI treatment.

In summary, we characterized the immune signature and cytokine milieu in women with POI and demonstrated that POI may result from a breakdown of immunological self‐tolerance evidenced by T_reg_ cell deficiency and consequently unrestrained immune destruction by an exacerbated T_H_1 response. These results provide new insights into the pathogenesis of POI and pave the way for novel therapeutic interventions for patients.

## 4 METHODS AND MATERIALS

### 4.1 Human subjects

All participants were recruited from the Center for Reproductive Medicine, Shandong University from October 2016 to November 2019. In total, patients with POI and biochemical POI (bPOI) and control women with normal ovarian reserve were selectively recruited. The inclusion criteria for POI included secondary amenorrhea for at least 4 months, serum basal FSH > 25 IU/L (on two occasions > 1 month apart) before age 40 according to the ESHRE and Chinese guideline.^1^, ^2^ BPOI, by some also called premature ovarian aging, was defined as regular or irregular menses and elevated basal serum FSH (10 IU/L < FSH ≤ 25 IU/L, on two occasions > 4 weeks apart) and antral follicle count (AFC) < 5 before age of 35 years old as previously reported.^49^, ^50^ Women with regular menstrual cycles and normal FSH level (<10 IU/L) sought for infertility treatment due to tubal obstruction or male factors were recruited as controls. Women with chromosomal abnormality, known gene mutations, history of ovarian surgery, radio‐or chemo‐therapy, history of recurrent spontaneous abortion, endometriosis or autoimmune disease, and infection in the previous three months, were excluded. The baseline characteristics are described in Tables S2 and S3. There are inevitable limitations which might confound the measurement of FF and granulosa cells, due to different controlled ovarian hyperstimulation protocols administrated based on different phenotypic characteristics of patients undergoing in vitro fertilization/ intracytoplasmic sperm injection and embryo transfer (IVF/ICSI‐ET). The human study was approved by the Institutional Review Board of Center for Reproductive Medicine, Shandong University. All participants had signed the written informed consent forms.

### 4.2 Mice

Female C57BL/6, B6AF1 and *Rag1* ^−/−^ mice (8‐ to 10‐week‐old) were obtained from The Jackson Laboratory. Foxp3^GFP‐Cre^ mice (on a C57BL/6 background) were bred in National Institutes of Health (NIH) facility (Bethesda, MD, USA). These mice were used for experimental POI models and housed in NIH facility. *Rag2* ^−/−^ mice purchased from Shanghai Model Organisms (Shanghai, China) and Foxp3^YFP‐Cre^ mice provided by Dr. B. Li (Shanghai Jiaotong University School of Medicine, Shanghai, China) were housed in animal facility of Experimental Animal Center of Shandong University (EAC‐SDU, Jinan, China), and used for some replication experiments. The immature female C57BL/6 mice (3‐week‐old) were purchased from EAC‐SDU for GCs isolation. All mice were housed in specific pathogen‐free conditions. All animal studies were performed according to NIH and SDU guidelines for the use and care of live animals and were approved by the Animal Care and Use Committees of the National Institute of Dental and Craniofacial Research (NIDCR), NIH and School of Medicine, Shandong University.

### 4.3 Hormone measurement and pelvic ultrasonography

Peripheral blood was sampled on day 2–4 of menstrual cycle or randomly (for women not menstruating frequently) in all patients and controls. Levels of basal FSH, luteinizing hormone, estradiol (E_2_), and total testosterone (T) were measured by chemiluminescence immunoassay (Roche Diagnostics, Mannheim, Germany). The intra‐assay and inter‐assay coefficients of variation were 10%. Transvaginal ultrasonography was routinely conducted and AFC was defined as the number of follicles 2–10 mm in early follicular phase.

### 4.4 Cell isolation

Human peripheral blood was collected to isolate PBMC using Ficoll‐Hypaque (MP Biomedicals, Santa Ana CA, USA) gradient centrifugation. Human GCs and FF were obtained from patients with bPOI and controls undergoing IVF/ICSI‐ET.

Murine lymphoid tissues (spleen and draining lymph nodes) were thoroughly minced and consecutively passed through the 70‐μm mesh strainer (BD Biosciences, San Jose, CA, USA) to obtain single‐cell suspensions. To prepare single cell suspension from ovary, ovaries from two mice were isolated, mixed and cut into small pieces, followed by enzymatic digestion for 20 minutes at 37°C in plain RPMI buffer (HyClone, Thermo Fisher Scientific, Waltham, MA, USA) with Collagenase IV (4 mg/ml; Gibco, Thermo Fisher Scientific) and DNase (0.01 mg/ml; Sigma, Louis, MO, USA), and then mashed through 70‐μm cell strainers.

Immature female C57BL/6 mice (3‐week‐old) were injected with 200 IU pregnant mare serum gonadotropin (PMSG, SANSHENG, Ningbo, Zhejiang, China) by intraperitoneal to stimulate follicle growth for 44 h. The ovaries were removed and primary GCs were isolated and harvested from large antral follicles by needle puncture.

### 4.5 ELISA assay for cytokines

The concentrations of different cytokines in human sera and FF were analyzed with a sandwich ELISA protocol. All the high‐sensitive kits were purchased from eBioscience (Thermo Fisher Scientific). The optical intensity was read at 450 nm as the primary wave length and 620 nm as the reference wave length on an automated microplate reader (Tecan, Switzerland). The 5‐parameter curve fit was used to determine the concentration of cytokines according to the manufacturer's instruction.

### 4.6 Flow cytometry

Dead cells were excluded from analysis using Zombie Yellow or Aqua Fixable Viability Kit (BioLegend, San Diego, CA, USA). For cell‐surface staining, 10^6^ cells per sample were incubated with various antibodies in staining buffer (phosphate‐buffered saline (PBS) and 2.5% fetal bovine serum) for 15 min at room temperature in the dark. For intranuclear staining, the cells were fixed and permeabilized in Fixation/Permeabilization buffer solution (eBioscience, Thermo Fisher Scientific) at 4°C for 1 h. For intracellular cytokine staining, cells were stimulated with Cell activation cocktail (BioLegend) at 37°C for 4 h, and then fixed and permeabilized with the Fixation/Permeabilization buffer solution (BD Biosciences) at 4°C for 20 min according to the manufacturer's instructions. Stained cells were acquired on an LSR Fortessa cell analyzer (BD Biosciences), and data were analyzed with FlowJo software (V 10.6.1, BD Biosciences, Ashland, OR, USA). The antibodies used are listed in Supplemental Table S4.

### 4.7 Adoptive transfer model of oophoritis and POI

*Rag1* ^–/–^ recipients were injected i.p. with FACS‐sorted naive CD4^+^CD25^–^CD45RB^hi^ T cells (4 × 10^5^ cells/mouse; BD FACSAria II) in the presence (POI+T_reg_ group) or absence (POI group) of FACS‐sorted CD4^+^CD25^hi^GFP^+^ T_reg_ cells (2.5 × 10^5^ cells/mouse; BD FACSAria II) from spleens and peripheral lymph nodes of Foxp3^GFP‐Cre^ mice. For replication, *Rag2* ^−/−^ and Foxp3^YFP‐Cre^ mice were used in some experiments. The *Rag1* ^–/–^ mice injected i.p. with PBS were included as controls. The recipient mice were weighted twice a week for 5 weeks. Serum was collected and isolated for estradiol and progesterone measurements. Ovarian tissues were harvested for histopathological and immunological analyses. Spleen and ovary draining lymph nodes were isolated for FACS analysis.

### 4.8 T_reg_ depletion in ZP3 induced POI model

Female B6AF1 mice received two doses of anti‐CD25 antibody (PC‐61.5.3, Bio X Cell, West Lebanon, NH, USA; 500 μg/mice; i.p.) on day 0 and 3 for T_reg_ depletion. Age‐matched control recipients were treated with isotype control antibody (HRPN, Bio X Cell, West Lebanon, NH, USA; 500 μg/mice; i.p.). All mice were immunized s.c. with 100 IU ZP3 peptide (amino acids 330–342, NSSSSQFQIHGPR, Invitrogen, Thermo Fisher Scientific) emulsified in complete Freund's Adjuvant (CFA, Sigma, Louis, MO, USA) on day 4 to induce autoimmune ovarian insufficiency. Mice were monitored weekly and sacrificed 3 weeks later. The ovaries were isolated for histological and functional analysis. Spleen and draining lymph nodes were used for FACS analysis.

### 4.9 Cell culture and cytokines treatment

As a steroidogenic human granulosa‐like tumor cell line, the KGN cell line obtained from RIKEN BioResource Center, Ibaraki, Japan^51^ was used for in vitro functional study. The KGN cells were cultured in DMEM/F12 medium (Thermo Fisher Scientific) containing 10% FBS (Biological Industries, Beit Haemek, Israel) and 1% penicillin/streptomycin (Thermo Fisher Scientific). Mice primary GCs were cultured in DMEM/F12 with 5% FBS. All cells were maintained in a humidified atmosphere containing 5% CO_2_ at 37°C.

Cells were treated in the absence or presence of recombinant human or murine IFN‐γ (PeproTech, Rocky Hill, NJ, USA), TNF‐α (PeproTech, Rocky Hill, NJ, USA), or a combination for 48 h. Then the cells were evaluated *in vitro* for cell proliferation and apoptosis assay. For steroid hormone measurements, cells were cultured in phenol‐red free DMEM/F12 medium (Thermo Fisher Scientific) containing 10% charcoal‐stripped FBS (Thermo Fisher Scientific). After 48 hours’ culture, the culture medium was supplemented with 10 nmol/ml testosterone as a substrate for estradiol generation for 24 h. The supernatant media was retained for estradiol measurement, and cells lysates were stored at −80°C until total RNA and protein extraction.

### 4.10 Cell proliferation and apoptosis assay

Cell proliferation was further carried out using Cell‐ Light EdU Apollo 567 In Vitro Imaging Kit (RiboBio, Guangzhou, China) according to the manufacturer's instructions. The rate of EdU‐positive cells was calculated with (EdU positive cells/Hoechst‐stained cells) × 100%. Cell apoptosis was detected by Annexin V/7‐AAD staining (BD Pharmingen, San Diego, CA, USA) and acquired on an LSR Fortessa (BD Biosciences, San Jose, CA, USA).

### 4.11 CTGF downregulation and human recombinant CTGF supplement

To explore the effect of *CTGF* on GCs function, shRNAs for *CTGF* (GenePharma Inc, Shanghai, China) were transfected at 50 nM using X‐ tremeGENE siRNA Transfection Reagent (Roche, Mannheim, Germany) to downregulate endogenous *CTGF* expression of KGN cells (sh‐CTGF). Non‐target shRNA (sh‐NC) was used as control. Additionally, KGN cells were incubation with different cytokines or in combination in the presence or absence of 20.0 ng/ml recombinant CTGF (R&D, Minneapolis, MN, USA.) to investigate the rescue effect of CTGF.

### 4.12 Pathway inhibitor or neutralizing antibody treatment

To further determine whether CTGF was directly regulated by IFN‐γ and TNF‐α, KGN cells were preincubated for 1 h with DMSO vehicle control, 10 μM AG‐490 (a selective JAK inhibitor, Selleck, Shanghai, China) or 5 μM Bay11‐7082 (an inhibitor of IKBα phosphorylation, Selleck), followed by addition of IFN‐γ and TNF‐α. Additionally, 1 μg/ml neutralizing antibodies of IFN‐γ (R&D) and TNF‐α (R&D) were added to block cytokines effects prior to cytokines stimulation.

### 4.13 Quantitative RT‐qPCR

Total RNA was isolated using TRIzolTM reagent (Ambion, Thermo Fisher Scientific), and cDNA was generated with Taq‐Man reverse transcription reagents (Applied Biosystems) or PrimeScript RT Reagent Kits (Takara Bio). Quantitative real‐time PCR was performed in triplicate using TaqMan gene expression assays (Applied Biosystems) or SYBR Premix Ex TaqTM Kit (Takara Bio) on 7500 real‐time PCR System (Applied Biosystems) or Roche LightCycle 480 (Roche). The level of target gene expression was quantified after normalization to *ACTB*, *Hprt* or *Gapdh* expression. The primers were listed in Table S5.

### 4.14 Western blot analysis

Total protein was harvested with RIPA buffer (Beyotime, Jiangsu, China) containing a phenylmethylsulfonyl fluoride (PMSF, Beyotime, Jiangsu, China) and phosphatase inhibitor cocktail (Cell Signaling Technology). Protein concentrations were determined using BCA assay (Pierce, Thermo Fisher Scientific). Primary antibodies recognizing CTGF, STAT1, Phospho‐STAT1, Phospho‐P65 (all 1:1000, Cell Signaling Technology), Phospho‐IKB‐α (1:500, Cell Signaling Technology), CYP19A1, PCNA (all 1:1000, Proteintech), and cleaved‐PARP (1:1000, Cell Signaling Technology) were incubated to examine the proteins. The α‐tubulin (1:2000, Proteintech) was used as an intrinsic control. The immunoreactive bands were photographed and analyzed with ChemiDoc MP Imaging System and Image Lab Software (Bio‐Rad, Hercules, CA, USA).

### 4.15 Histology and immunohistochemistry staining

Bouin‐fixed, paraffin‐embedded ovarian tissue was serially sections at 5 μm in thickness. Every fifth section was analyzed for the presence of oocytes and follicles after the H&E staining as previously described. The counting results were multiplied by five to estimate the total numbers of oocytes and follicles in each ovary. For immunohistochemistry, the sections of mice ovaries were stained with anti‐cleaved‐PARP (1:100, Cell Signaling Technology). The image was taken with microscope (OLYMPUS, Japan) and analyzed by ImageJ software (NIH, Bethesda, MD, USA).

### 4.16 Statistical analysis

All experiments were independently repeated for three times and performed in triplicate. Statistical analysis was performed using SPSS version 21 (SPSS Inc., Chicago, IL, USA) and GraphPad Prism 7 (San Diego, USA). When continuous data was normality distributed, it was shown as Mean ± standard error of the mean (SEM) and determined with the two‐tailed Student's *t* test or one‐way ANOVA test; otherwise data were expressed as median (quartile) and compared by two‐tailed Mann‐Whitney U test. Categorical variables were analyzed with chi‐square test. Spearman's correlation was used to estimate the association between immune indicators and biomarkers of ovarian reserve. *p* < 0.05 was considered significant.

## CONFLICT OF INTEREST

The authors have declared that no conflict of interest exists.

## AUTHOR CONTRIBUTIONS

Xue Jiao and Xiruo Zhang designed and performed most of the experiments, analyzed and interpreted the data, and contributed to the writing of the manuscript. Nianyu Li, Dunfang Zhang, Shidou Zhao, and Yujie Dang performed experiments, analyzed data, and contributed to the writing of the manuscript. Peter Zanvit and Wenwen Jin performed experiments and provided support and/or critical scientific input. Zi‐Jiang Chen, Wanjun Chen, and Yingying Qin conceived of and directed the research, designed the experiments, and wrote the paper.

## Supporting information

Supporting information

Supporting information
