# Supplementary material for: Treg deficiency‐mediated TH1 response causes human premature ovarian insufficiency through apoptosis and steroidogenesis dysfunction of granulosa cells
Source: Clin Transl Med. 2021 Jun 20;11(6):e448. doi: 10.1002/ctm2.448 (PMC8214854; doi:10.1002/ctm2.448)
Supplement: Supplementary file 1 — Supporting information [file CTM2-11-e448-s002.pdf]

Supplemental Figure S1

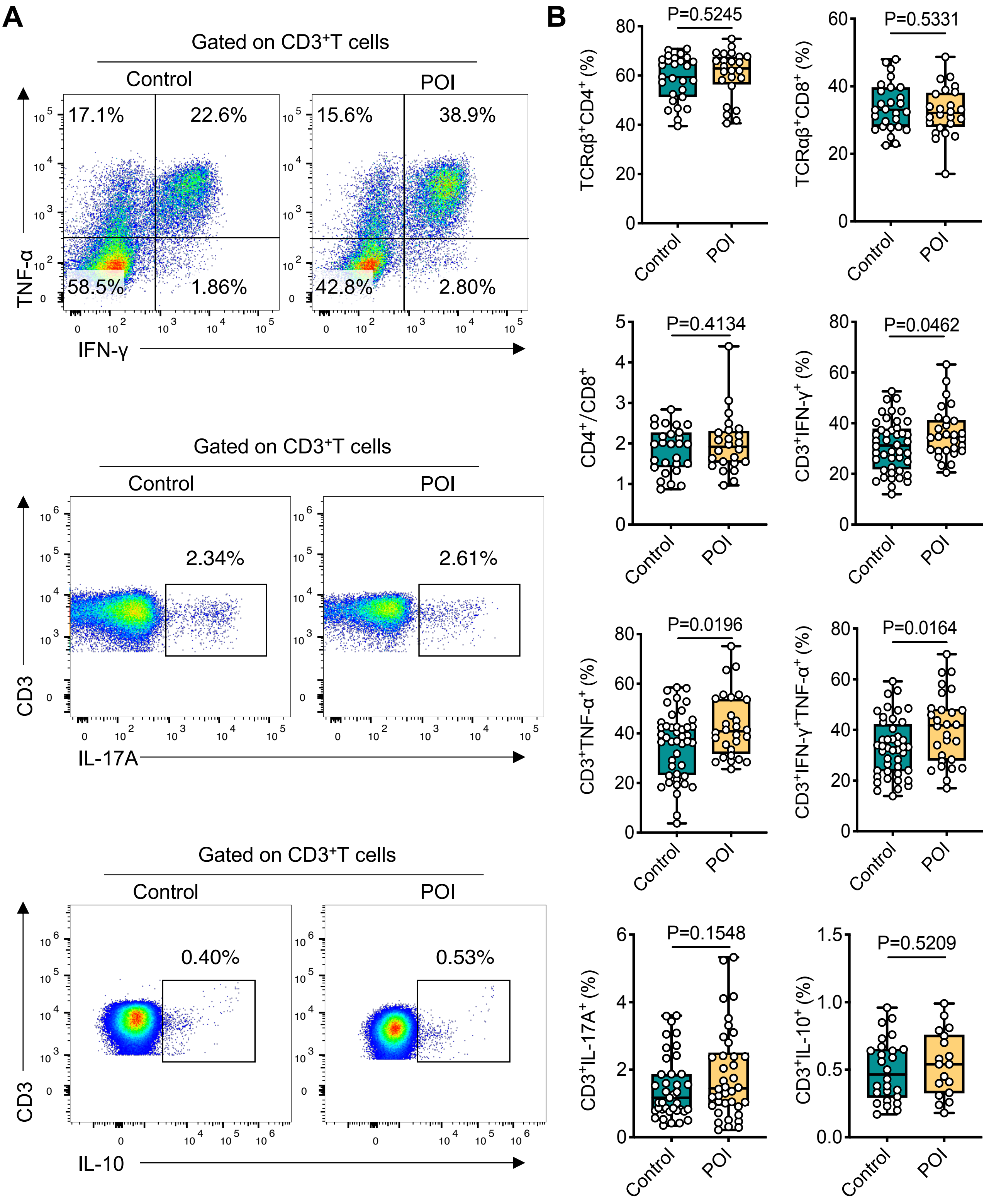

Supplemental Figure S1. Distribution of different T lymphocyte subsets in peripheral blood mononuclear cell (PBMC) in patients with POI and controls.

(A) Representative flow cytometry plots and (B) statistics of different T lymphocyte subsets in $CD3^+$ T cells in control subjects (N=44) and patients with POI (N=28). Data were shown as box-and-whisker plots and analyzed by unpaired two-tailed Student’s t-test. Dots represent individual data points.

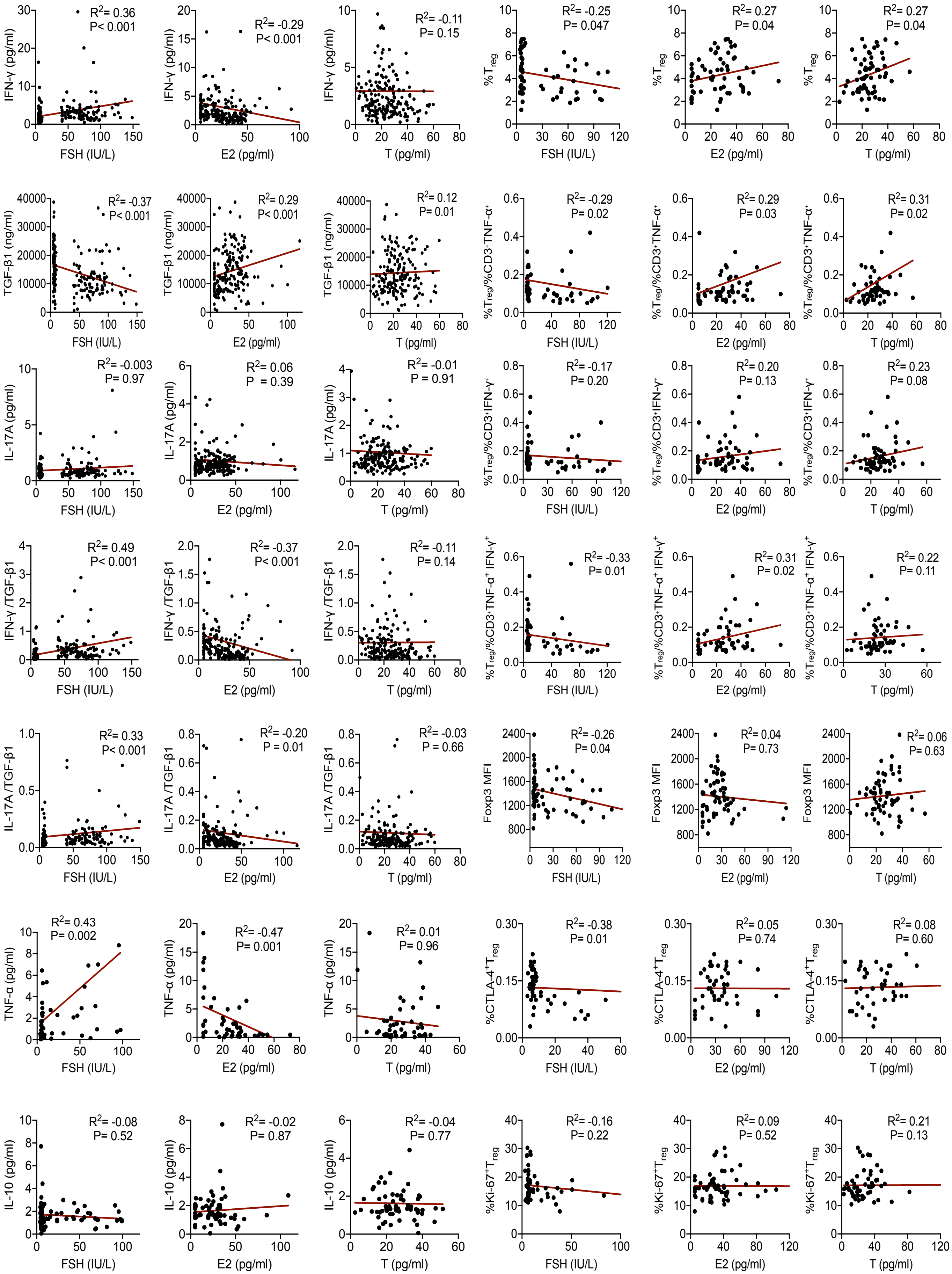

IFN-γ (pg/ml)
R2= 0.36
P< 0.001
FSH (IU/L)
IFN-γ (pg/ml)
R2= -0.29
P< 0.001
E2 (pg/ml)
IFN-γ (pg/ml)
R2= -0.11
P= 0.15
T (pg/ml)
%Treg
R2= -0.25
P= 0.047
FSH (IU/L)
%Treg
R2= 0.27
P= 0.04
E2 (pg/ml)
%Treg
R2= 0.27
P= 0.04
T (pg/ml)
TGF-β1 (ng/ml)
R2= -0.37
P< 0.001
FSH (IU/L)
TGF-β1 (ng/ml)
R2= 0.29
P< 0.001
E2 (pg/ml)
TGF-β1 (ng/ml)
R2= 0.12
P= 0.01
T (pg/ml)
%Treg/%CD3+TNF-α+
R2= -0.29
P= 0.02
FSH (IU/L)
%Treg/%CD3+TNF-α+
R2= 0.29
P= 0.03
E2 (pg/ml)
%Treg/%CD3+TNF-α+
R2= 0.31
P= 0.02
T (pg/ml)
IL-17A (pg/ml)
R2= -0.003
P= 0.97
FSH (IU/L)
IL-17A (pg/ml)
R2= 0.06
P = 0.39
E2 (pg/ml)
IL-17A (pg/ml)
R2= -0.01
P = 0.91
T (pg/ml)
%Treg/%CD3+IFN-γ+
R2= -0.17
P= 0.20
FSH (IU/L)
%Treg/%CD3+IFN-γ+
R2= 0.20
P= 0.13
E2 (pg/ml)
%Treg/%CD3+IFN-γ+
R2= 0.23
P= 0.08
T (pg/ml)
IFN-γ /TGF-β1
R2= 0.49
P< 0.001
FSH (IU/L)
IFN-γ /TGF-β1
R2= -0.37
P< 0.001
E2 (pg/ml)
IFN-γ /TGF-β1
R2= -0.11
P= 0.14
T (pg/ml)
%Treg/%CD3+TNF-α+ IFN-γ+
R2= -0.33
P= 0.01
FSH (IU/L)
%Treg/%CD3+TNF-α+ IFN-γ+
R2= 0.31
P= 0.02
E2 (pg/ml)
%Treg/%CD3+TNF-α+ IFN-γ+
R2= 0.22
P= 0.11
T (pg/ml)
IL-17A /TGF-β1
R2= 0.33
P< 0.001
FSH (IU/L)
IL-17A /TGF-β1
R2= -0.20
P = 0.01
E2 (pg/ml)
IL-17A /TGF-β1
R2= -0.03
P = 0.66
T (pg/ml)
Foxp3 MFI
R2= -0.26
P= 0.04
FSH (IU/L)
Foxp3 MFI
R2= 0.04
P= 0.73
E2 (pg/ml)
Foxp3 MFI
R2= 0.06
P= 0.63
T (pg/ml)
TNF-α (pg/ml)
R2= 0.43
P= 0.002
FSH (IU/L)
TNF-α (pg/ml)
R2= -0.47
P= 0.001
E2 (pg/ml)
TNF-α (pg/ml)
R2= 0.01
P= 0.96
T (pg/ml)
%CTLA-4+Treg
R2= -0.38
P= 0.01
FSH (IU/L)
%CTLA-4+Treg
R2= 0.05
P= 0.74
E2 (pg/ml)
%CTLA-4+Treg
R2= 0.08
P= 0.60
T (pg/ml)
IL-10 (pg/ml)
R2= -0.08
P= 0.52
FSH (IU/L)
IL-10 (pg/ml)
R2= -0.02
P= 0.87
E2 (pg/ml)
IL-10 (pg/ml)
R2= -0.04
P= 0.77
T (pg/ml)
%Ki-67+Treg
R2= -0.16
P= 0.22
FSH (IU/L)
%Ki-67+Treg
R2= 0.09
P= 0.52
E2 (pg/ml)
%Ki-67+Treg
R2= 0.21
P= 0.13
T (pg/ml)

Supplemental Figure S2. Correlation between immune indicators in periphery with biomarkers of ovarian reserve (FSH, E2 and T).

Data were analyzed by Spearman's correlation.
