# Supplementary material for: Treg deficiency‐mediated TH1 response causes human premature ovarian insufficiency through apoptosis and steroidogenesis dysfunction of granulosa cells
Source: Clin Transl Med. 2021 Jun 20;11(6):e448. doi: 10.1002/ctm2.448 (PMC8214854; doi:10.1002/ctm2.448)
Supplement: Supplementary file 2 — Supporting information [file CTM2-11-e448-s001.docx]

**Supplemental Table S1. Correlation between immune indicators in ovarian microenvironment with biomarkers of ovarian reserve.**

| **Variables** | **FSH** | | **E2** | | **T** | |
| --- | --- | --- | --- | --- | --- | --- |
|  | R | P | R | P | R | P |
| FF TNF-α | -0.02 | 0.87 | 0.04 | 0.74 | -0.09 | 0.44 |
| FF IL-10 | -0.18 | 0.20 | 0.32 | 0.02 | 0.00 | 0.98 |
| FF TNF-α/IL-10 | -0.26 | 0.07 | 0.31 | 0.02 | 0.19 | 0.17 |
| GC *IFNG* | -0.08 | 0.57 | 0.09 | 0.50 | 0.16 | 0.22 |
| GC *TGFB1* | -0.15 | 0.35 | 0.33 | 0.04 | 0.13 | 0.41 |
| GC *IL17A* | 0.10 | 0.44 | -0.20 | 0.13 | 0.04 | 0.75 |
| GC *TNF* | 0.24 | 0.056 | -0.06 | 0.64 | -0.17 | 0.17 |
| GC *IL10* | -0.17 | 0.36 | 0.19 | 0.30 | 0.19 | 0.29 |
| GC *IFNG/TGFB1* | -0.15 | 0.38 | 0.12 | 0.49 | 0.16 | 0.35 |
| GC *IFNG/IL10* | 0.08 | 0.66 | 0.00 | 0.99 | -0.01 | 0.97 |
| GC *TNF/IL10* | 0.16 | 0.41 | -0.43 | 0.02 | 0.02 | 0.94 |
| GC *TNF/TGFB1* | 0.10 | 0.56 | -0.18 | 0.29 | -0.06 | 0.74 |

FSH: Follicle stimulating hormone; E2: Estradiol; T: testosterone; FF: Follicular fluid; GC: Granulosa cells. Data were analyzed by Spearman’s correlation.

**Supplemental Table S2. Clinical characteristic of patients with POI and control wom****en.**

| **Variables** | **POI** | **Control** | **P-value** |
| --- | --- | --- | --- |
| Age (yrs) | 29.49 ± 0.38 | 30.46 ± 0.28 | 0.04 |
| BMI (kg/m^2^) | 22.70 ± 0.26 | 23.01 ± 0.28 | 0.42 |
| FSH (IU/L) | 61.60 ± 2.34 | 6.29 ± 0.10 | <0.0001 |
| E2 (pg/mL) | 24.25 ± 2.51 | 35.01 ± 1.13 | <0.0001 |
| T (ng/dL) | 22.61 ± 1.21 | 27.54 ± 1.08 | 0.0032 |
| AMH (ng/mL) | 0.08 (0.06, 0.09) | 4.05 (2.29, 5.92) | <0.0001 |
| TSH (μIU/mL) | 2.22 ± 0.10 | 2.29 ± 0.06 | 0.59 |

BMI: Body mass index; FSH: Follicle stimulating hormone; E2: Estradiol; T: testosterone; AMH: anti- müllerian hormone; TSH：Thyroid stimulating hormone. Data were expressed as mean ± SEM or median (quartile), and were analyzed by unpaired two-tailed Student’s t-test or two-tailed Mann-Whitney U-test.

**Supplemental Table S3. Clinical characteristic of patients with biochemical POI (bPOI) and control women.**

| **Variables** | **bPOI** | **Control** | **P-value** |
| --- | --- | --- | --- |
| Age (yrs) | 32.29 ± 0.65 | 30.34 ± 0.57 | 0.03 |
| BMI (kg/m^2^) | 22.86±0.56 | 22.16±0.30 | 0.27 |
| FSH (IU/L) | 13.06 ± 0.55 | 6.54 ± 0.21 | <0.0001 |
| E2 (pg/mL) | 36.88 ± 3.21 | 45.42 ±5.51 | 0.19 |
| T (ng/dL) | 19.59 ± 1.85 | 20.87 ± 1.69 | 0.61 |
| AMH (ng/mL) | 0.64 (0.29, 1.10) | 3.03 (2.15, 5.05) | <0.0001 |
| TSH (μIU/mL) | 2.23 ± 0.20 | 1.90 ± 0.15 | 0.20 |
| No. of oocyte retrieved | 4.00 (2.00, 5.00) | 10.00 (8.00, 14.00) | <0.001 |
| E2 on HCG day (pg/mL) | 1350.00 (1006.45, 2036.50) | 2767.50 (2262.75, 3298.75) | <0.001 |
| P on HCG day (ng/mL) | 0.68 (0.43, 0.92) | 0.83 (0.65, 1.10) | 0.078 |
| Total dosage of Gn (IU) | 2075.00 (1200.00, 2962.50) | 1650.00 (1200.00, 2262.50) | 0.023 |

BMI: Body mass index; FSH: Follicle stimulating hormone; E2: Estradiol; T: testosterone; AMH: anti- müllerian hormone; TSH：Thyroid stimulating hormone. Data were expressed as mean ± SEM or median (quartile), and were analyzed by unpaired two-tailed Student’s t-test or two-tailed Mann-Whitney U-test.

**Supplemental Table S4. Primers used for qRT-PCR and CTGF silencing.**

|  | **Antibodies** | **Clone** | **Source** |
| --- | --- | --- | --- |
| **Human** | CD3 Alexa Fluor® 700 | OKT3 | eBioscience |
|  | CD3 APC-H | SK7 | BD Biosciences |
|  | CD4 PerCP-Cyanine5.5 | OKT4 | eBioscience |
|  | CD8 APC-Cy™7 | RPA-T8 | BD Biosciences |
|  | CD25 APC | BC96 | eBioscience |
|  | CD25 Brilliant Violet 421™ | BC96 | Biolegend |
|  | CD127 PE | A019D5 | Biolegend |
|  | Foxp3 PE | PCH101 | eBioscience |
|  | Foxp3 PE | 206D | Biolegend |
|  | TNF-α FITC | MAb11 | eBioscience |
|  | IFN-γ PE | 4S.B3 | eBioscience |
|  | IL-10 PE-Cyanine7 | JES3-9D7 | eBioscience |
|  | IL-17A APC | eBio64DEC17 | eBioscience |
|  | CTLA-4 Brilliant Violet 421™ | BNI3 | Biolegend |
|  | GITR Alexa Fluor® 488 | 108-17 | Biolegend |
|  | Ki-67 PE-Cyanine7 | 20Raj1 | eBioscience |
| **Mice** | CD45 Alexa Fluor® 700 | 30-F11 | eBioscience |
|  | CD45 APC-Cyanine7 | RA3-6B2 | eBioscience |
|  | TCR beta APC-eFluor 780 | H57-597 | eBioscience |
|  | CD4 Brilliant Violet 421 | RM4-5 | Biolegend |
|  | CD4 PerCP-Cyanine5.5/Alexa Fluor 700 | RM4-5 | eBioscience |
|  | CD8 PerCP-Cyanine5.5 | 53-6.7 | Biolegend |
|  | CD25 APC/PE | 3C7 | Biolegend |
|  | CD45RB PE | C363-16A | Biolegend |
|  | IFN-γ Brilliant Violet 421™ | XMG1.2 | Biolegend |
|  | IFN-γ eFluor 450 | XMG1.2 | eBioscience |
|  | TNF-α Brilliant Violet 650 | MP6-XT22 | BD Biosciences |
|  | TNF-α PE/FITC | MP6-XT22 | eBioscience |
|  | IL-17A APC/PE/PE-Cyanine7 | eBio17B7 | eBioscience |
|  | Foxp3 Alexa Fluor 647 | 3G3; | eBioscience |
|  | Foxp3 eFluor 450 | FJK-16s | eBioscience |

**Supplemental Table S5. Primers used for qRT-PCR and CTGF silencing.**

|  | **Gene** | **Forward Primer** | **Reverse Primer** |
| --- | --- | --- | --- |
| **Human** | *ACTB* | CATGTACGTTGCTATCCAGGC | CTCCTTAATGTCACGCACGAT |
|  | *IFNG*  *TNF* | TGATGGCTGAACTGTCGCCAGC  GAGGCCAAGCCCTGGTATG | CTGGGATGCTCTTCGACCTCGA  CGGGCCGATTGATCTCAGC |
|  | *IL17A*  *IL10* | TCCCACGAAATCCAGGATGC  TGGGGGAGAACCTGAAGAC | GGATGTTCAGGTTGACCATCAC  ACAGGGAAGAAATCGATGACA |
|  | *TGFB1* | GCAGCACGTGGAGCTGTA | CAGCCGGTTGCTGAGGTA |
|  | *IL4* | TGCCTCCAAGAACACAACTGA | CCAACGTACTCTGGTTGGCTT |
|  | *CYP19A1* | ACTACAACCGGGTATATGGAGAA | TCGAGAGCTGTAATGATTGTGC |
|  | *CTGF* | CAGCATGGACGTTCGTCTG | AACCACGGTTTGGTCCTTGG |
|  | *INHBA* | ACGGGTATGTGGAGATAGAGGA | GGACTTTTAGGAAGAGCCAGACT |
|  | *WT1* | CACAGCACAGGGTACGAGAG | CAAGAGTCGGGGCTACTCCA |
|  | *FOXO1* | TCGTCATAATCTGTCCCTACACA | CGGCTTCGGCTCTTAGCAAA |
|  | *GATA6* | CTGCGGGCTCTACAGCAAG | GTTGGCACAGGACAATCCAAG |
| **Mice** | *Gapdh* | accacagtccatgccatcac | tccaccaccctgttgctgta |
|  | *Ctgf* | gggcctcttctgcgatttc | atccaggcaagtgcattggta |
|  | *Ifng* | atgaacgctacacactgcatc | ccatccttttgccagttcctc |
|  | *Tnf* | gacgtggaactggcagaagag | ttggtggtttgtgagtgtgag |
|  | *IL1b* | gaaatgccaccttttgacagtg | tggatgctctcatcaggaca |
|  | *Ccr1* | gatttcacagaagcctacccca | aggttgaacaggtagatgctgg |
|  | *Ccr2* | aggagccatacctgtaaatgcc | ccgtggatgaactgaggtaaca |
|  | *Cxcl10* | aggggagtgatggagagagg | tgaaagcgtttagccaaaaaagg |
|  | *Cyp11a* | cgatactcttctcatgcgag | ctttcttccaggcatctgaac |
|  | *Cyp19a1* | aaccccatgcagtataatgtcac | aggacctggtattgaagacgag |
|  | *Fshr* | ccttgctcctggtctccttg | ctcggtcaccttgctatcttg |
|  | *Inha* | TaqMan primer, Mm00439683_m1 | Applied Biosystems |
|  | *Il17a* | TaqMan primer, Mm00439618_m1 | Applied Biosystems |
|  | *Amh* | TaqMan primer, Mm00431795_g1 | Applied Biosystems |
|  | *Hprt* | TaqMan primer, Mm00446968_m1 | Applied Biosystems |
| **CTGF silence** |  | Sense primer | Antisense primer |
|  | sh-NC | UUCUCCGAACGUGUCACGUTT | ACGUGACACGUUCGGAGAATT |
|  | sh-CTGF | GCUAAAUUCUGUGGAGUAUTT | AUACUCCACAGAAUUUAGCTT |
